# Genetic drift and selection in many-allele range expansions

**DOI:** 10.1371/journal.pcbi.1005866

**Published:** 2017-12-01

**Authors:** Bryan T. Weinstein, Maxim O. Lavrentovich, Wolfram Möbius, Andrew W. Murray, David R. Nelson

**Affiliations:** 1 School of Engineering and Applied Sciences, Harvard University, Cambridge, Massachusetts, United States of America; 2 Department of Physics and Astronomy, University of Pennsylvania, Philadelphia, Pennsylvania, United States of America; 3 Living Systems Institute, University of Exeter, Exeter, United Kingdom; 4 Physics and Astronomy, College of Engineering, Mathematics and Physical Sciences, University of Exeter, Exeter, United Kingdom; 5 Department of Physics, Harvard University, Cambridge, Massachusetts, United States of America; 6 FAS Center for Systems Biology, Harvard University, Cambridge, Massachusetts, United States of America; 7 Department of Molecular and Cellular Biology, Harvard University, Cambridge, Massachusetts, United States of America; MIT, UNITED STATES

## Abstract

We experimentally and numerically investigate the evolutionary dynamics of four competing strains of *E. coli* with differing expansion velocities in radially expanding colonies. We compare experimental measurements of the average fraction, correlation functions between strains, and the relative rates of genetic domain wall annihilations and coalescences to simulations modeling the population as a one-dimensional ring of annihilating and coalescing random walkers with deterministic biases due to selection. The simulations reveal that the evolutionary dynamics can be collapsed onto master curves governed by three essential parameters: (1) an expansion length beyond which selection dominates over genetic drift; (2) a characteristic angular correlation describing the size of genetic domains; and (3) a dimensionless constant quantifying the interplay between a colony’s curvature at the frontier and its selection length scale. We measure these parameters with a new technique that precisely measures small selective differences between spatially competing strains and show that our simulations accurately predict the dynamics without additional fitting. Our results suggest that the random walk model can act as a useful predictive tool for describing the evolutionary dynamics of range expansions composed of an arbitrary number of genotypes with different fitnesses.

## Author summary

Population expansions occur naturally during the spread of invasive species and have played a role in our evolutionary history when humans migrated out of Africa. We use a colony of non-motile bacteria expanding into unoccupied, nutrient-rich territory on an agar plate as a model system to explore how an expanding population’s spatial structure impacts its evolutionary dynamics. Spatial structure is present in expanding microbial colonies because daughter cells migrate only a small distance away from their mothers each generation. Generally, the constituents of expansions occurring in nature and in the lab have different genetic compositions (genotypes, or alleles if a single gene differs), each instilling different fitnesses, which compete to proliferate at the frontier. Here, we show that a random-walk model can accurately predict the dynamics of four expanding strains of *E. coli* with different fitnesses; each strain represents a competing allele. Our results can be extended to describe any number of competing genotypes with different fitnesses in a naturally occurring expansion as long as the underlying motility of the organisms does not cause our model to break down. Our model can also be used to precisely measure small selective differences between spatially competing genotypes in controlled laboratory settings.

## Introduction

A competition between stochastic and deterministic effects underlies evolution. In a well-mixed system such as a shaken culture of the yeast microorganism *Saccharomyces cerevisiae*, stochastic competition between individuals, mutations, and selection dictate the dynamics of the population [1]. In spatially structured environments, active or passive dispersal of individuals also plays an important role. The local “well-mixed” dynamics must be coupled to the motion of individuals, leading to strikingly different evolutionary dynamics, even in the absence of selection [2–7].

**Fig 1:**
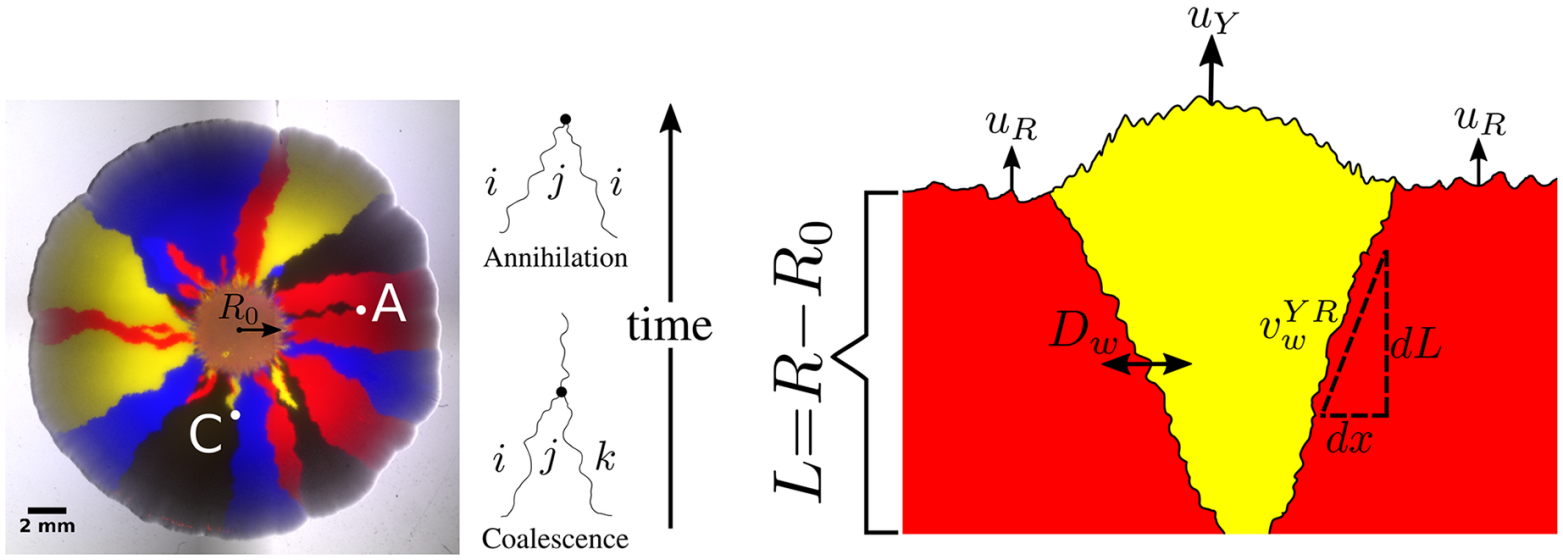
*Left*: A four-color *E. coli* range expansion. Four strains of *E. coli* differing only by a heritable fluorescent marker were inoculated on an agar plate in a well-mixed droplet and expanded outwards, leaving behind a “frozen record” of their expansion. Virtually all growth occurred at the edge of the colony. The markers instilled different expansion velocities: our eCFP (blue) and eYFP (yellow) strains expanded the fastest, followed by our black strain, and finally our mCherry (red) strain. As a result of the differing expansion velocities, the yellow/blue bulges at the frontier are larger than the black bulges which are larger than the red bulges, although the significant stochastic undulations at the front mask their size. The microbes segregate into one color locally at a critical expansion radius *R*_0_ due to extreme genetic drift at the frontier [8]. After segregated domains form, genetic domain walls diffuse and collide with neighboring walls in an “annihilation” or “coalescence” event indicated by an *A* or *C*, respectively. *Right:* Illustration of the relevant parameters used to model range expansions. Here, a faster expanding, more fit yellow strain with expansion velocity *u*_*Y*_ is sweeping through a less fit red strain with expansion velocity *u*_*R*_, in a regime where the curvature of the colony can be neglected. The length expanded by the colony is *L* = *R* − *R*_0_. We characterize domain wall motion per differential length expanded *dL* and the wall’s differential displacement perpendicular to the expansion direction *dx*. $v_{w}^{YR}$ is a dimensionless speed, characterizing the yellow-red (*YR*) domain wall’s average expansion *dx* per length expanded *dL*, i.e., $v_{w}^{YR}=dx/dL.$ *D_w_* is the domain walls’ diffusion coefficient per length expanded; it controls how randomly the domain walls move. We treat the dynamics of our four strains as a one-dimensional line of annihilating and coalescing random walkers using the parameters *R*_0_, *D*_*w*_, and $v_{w}^{ij}$, where *ij* represents all possible domain wall types.

A model laboratory system that can be used to explore the coupling between local “well-mixed” effects and spatial deterministic and stochastic dynamics is a microbial range expansion [8], in which a population expands into an unoccupied region of a hard agar Petri dish. Non-motile microbes expand outwards from their initial position due to a combination of growth coupled with random pushing by neighboring cells and leave behind a record of their genetic competition as they cannot move and cease reproducing once the population becomes too dense [8]. A frozen genetic pattern of four competing strains of *E. coli* marked by different fluorescent colors can be seen in Fig 1. Spatial structure is present in the frozen genetic patterns because the microbes at the expanding frontier produce daughter cells of the same color that migrate only a small fraction of the front circumference within a generation. Hallatschek et al. [8] identified the key role of genetic drift in producing these sectored patterns; the small population size at the front of an expanding population [9, 10] enhances number fluctuations (i.e. genetic drift), eventually leading to the local fixation of one strain past a critical expansion radius *R*_0_. The decrease in genetic diversity as the small number of individuals at the frontier expands is referred to as the “Founder effect” [11].

Outside of the laboratory, range expansions occur naturally during the spread of invasive species such as the bank vole in Ireland [12] or the cane toad in Australia [13], and played a role in the evolutionary history of humans when migrating out of Africa [14]. In these natural expansions, populations may have many competing genotypes, or alleles, each instilling a different fitness. Even if a population is originally clonal, mutations may create new alleles that compete with one another to proliferate, a phenomenon known as clonal interference [15].

An allele’s fitness is often determined by its corresponding expansion velocity. Faster expanding individuals will colonize more territory and will block slower strains from expanding, resulting in the increased abundance of ‘faster’ alleles at the frontier [13, 16, 17]. If the curvature of a microbial colony can be neglected and its front is sufficiently smooth, it has been shown both theoretically and experimentally that the domain wall of a faster expanding strain will displace a slower expanding strain at a constant rate per length expanded after an initial transient, resulting in a characteristic triangular shape [17] as shown on the right side of Fig 1. If the curvature of the expansion is not negligible, the sector boundaries will trace logarithmic spirals [17].

Even in the most simple scenario when de-novo mutations and mutualistic or antagonistic interactions are ignored, the dynamics of *many* competing alleles with varying fitnesses at the front of a range expansion have neither been quantified theoretically nor explored in laboratory experiments. Prior laboratory experiments focused on the dynamics of a single sector of a more fit strain (representing a competing alelle) of yeast sweeping through a less fit strain [17] in regimes where stochastic wandering of genetic boundaries was not expected to be important. Recent experimental work studied how fast a *single* more fit strain swept through a less fit strain in a range expansion and compared the dynamics to the same strains in a well mixed test tube [9].

In this paper, we experimentally and numerically investigate the dynamics of four competing strains (alleles) of *E. coli* with varying selective advantages initially distributed randomly at the front of a radial range expansion. The eCFP (blue) and eYFP-labeled (yellow) strains expanded the fastest, followed by the non-fluorescent (black) strain, and finally the mCherry-labeled (red) strain. The differences in expansion speeds are reflected in Fig 1 as follows: the yellow/blue bulges at the front of the expansion are larger than the black bulges which are larger than the red bulges. The significant random undulations at the frontier, however, significantly mask the selection-induced bulges.

As is evident from Fig 1, the size and location of a monoclonal sector can be described by the locations of its boundaries. When two boundaries collide, they either *annihilate* if the neighbors to the left and right of the collision are the same or *coalesce* if the neighbors are different, as illustrated by the *A* and *C* respectively on the left side of Fig 1. We therefore describe our expansions as a one-dimensional line of annihilating and coalescing random walkers, a description that has been used extensively in previous work (see Ref. [2] for a review). To account for the radial geometry of our colonies, we allow the frontier to inflate, corresponding to the increasing perimeter of the colony as its radius increases. Past the radius *R*_0_ where genetic domains originally form, we describe the random motion of genetic domains by a diffusion constant per length expanded *D*_*w*_ (see Fig 1) [18]. If *dx* characterizes the displacement of a domain wall perpendicular to the expansion direction and *dL* is the distance the colony has expanded (the radius that the colony has grown) as illustrated on the right side of Fig 1, where we neglect the circumferential curvature in this small region, we define the diffusion constant per length expanded as 2*D*_*w*_ = *d*Var(*x*)/*dL* where Var(*x*) ≡ 〈*x*^2^〉 − 〈*x*〉^2^ is the variance and the brackets indicate an average over many domain walls. Note that *D*_*w*_ has dimensions of length. Similarly, differences in expansion velocities between neighboring strains will lead to the deterministic displacement of domain walls per length expanded as the faster expanding strain will reach the contested point on the front before a slower growing strain as mentioned above [17]; we characterize this deterministic motion by a dimensionless “wall velocity,” [18] $v_{w}^{ij}=d\langle x\rangle /dL$, where *i* is the strain to the left of the domain wall and *j* is the strain to the right. Note that $v_{w}^{ij}=-v_{w}^{ji}$.

The dynamics of an arbitrary number of *neutral* competing strains in an expansion (i.e. $v_{w}^{ij}=0$ for all domain walls) is well understood as the dynamics can be described as a one-dimensional system of annihilating and coalescing random walkers [19–21] which is equivalent to a one-dimensional *q*-state Potts model [22, 23] governed by zero-temperature Glauber dynamics [24] or a *q*-opinion Voter model [25, 26]. Many theoretical predictions and analyses of this system exist; of particular relevance to this paper are the relative annihilation and coalescence rates per collision as *q* is varied [27–29] and the calculation of spatial correlation functions [28]. To map standard linear results onto an inflating ring (i.e. including *R*_0_ in the models), one can use a conformal time transformation [30–32]. Fewer results are available in the presence of selection, i.e. when domain walls have deterministic biases (nonzero $v_{w}^{ij}$) [33]. Analytical results are rare because the moment hierarchy of this model does not close [2] as discussed in S1 Appendix.

In this paper, we measure and predict three quantities relevant to the evolutionary dynamics of our four competing strains of *E. coli* in radial range expansions: the average fraction of each of our four strains, the two-point correlation functions between our strains, and the relative annihilation and coalescence probabilities per domain wall collision (see Fig 1), a quantity that has received theoretical attention [27–29] but has neither been explored experimentally nor investigated in the presence of selection. We measure these three quantities using an image analysis toolkit (available on GitHub, complete with examples of how to use it [34]) that extends experimental techniques for two-color (two-allele) range expansions [8, 9, 17, 18, 35, 36] to an arbitrary number of competing strains. We next use an efficient radial simulation (also on GitHub [34]) of annihilating and coalescing random walkers with deterministic wall velocities to determine what sets the scale of the dynamics and to synthesize our experimental and theoretical results. We show that three key combinations of *R*_0_, *D*_*w*_ and $v_{w}^{ij}$ control the dynamics of our four strains. We conclude with suggestions for future studies. The details of our experimental, theoretical, and simulation methods are given in the last section.

## Results

### Experimental results

We begin by reporting our measurements of the average fraction of each strain, the two-point correlation functions between strains, and the relative rates of annihilations and coalescences as a function of length expanded for our four competing strains of *E. coli*. As discussed in the Materials and Methods, we found that our eCFP and eYFP strains had the fastest expansion velocities followed by the black strain and finally the mCherry strain (see Table 1). We expected that our experimental measurements would reflect this hierarchy of speeds; faster expanding strains should have a larger fitness than slower expanding ones. To illustrate the presence of selection, we used neutral theory (discussed in detail in S1 Appendix) as a null expectation; selection caused deviations from the neutral predictions. To calibrate neutral theory to our experiments we fit *R*_0_ and *D*_*w*_, two model parameters illustrated in Fig 1, following the procedures discussed in the Materials and Methods. The fit values of *R*_0_ and *D*_*w*_ can be seen in Table 2. In later sections, we show how to predict the average fraction, two-point correlation functions, and relative rates of annihilation and coalescences using our random-walk model and simulation.

**Table 1:** The expansion velocity *u*_*i*_ and each strain’s selective advantage relative to the mCherry strain *s*_*iR*_ = *u*_*i*_/*u*_*R*_ − 1 measured over the course of seven days for radii greater than *R*_0_ (the radius where distinguishable domain walls formed) averaged over three independent experiments conducted on separate sets of agar plates. $s_{iR}^{wm}=g_{i}/g_{R}-1$ was the fitness of each strain relative to mCherry in *liquid* culture with respect to their basal growth rates *g*_*i*_ and *g*_*R*_. The radial expansion velocity fitness *s*_*iR*_ did not match the well-mixed liquid-culture fitness $s_{iR}^{wm}$. However, every strain in liquid culture still grew faster than mCherry. Interestingly, the black strain grew faster than the eCFP and eYFP strains in liquid culture while on agar, the eCFP and eYFP strains expanded faster than the black strain. See the Materials and methods for additional information.

Strain	Average Velocity *u*_*i*_ (mm/day)	*s*_*iR*_ = *u*_*i*_/*u*_*R*_ − 1	$s_{iR}^{wm}=g_{i}/g_{R}-1$
eYFP	1.18 ± 0.02	0.09 ± 0.03	0.018 ± 0.003
eCFP	1.17 ± 0.02	0.09 ± 0.03	0.018 ± 0.003
Black	1.14 ± 0.02	0.06 ± 0.01	0.037 ± 0.003
mCherry	1.07 ± 0.02	0	0

**Table 2:** Parameters used in the annihilating and coalescing random-walk model. We experimentally measured *R*_0_, *D*_*w*_, and $v_{w}^{ij}$ using the procedures outlined in the Materials and methods so that we could compare experimental results with our model’s predictions.

Parameter	Value	Description
Experimentally Measured		
*R*_0_	3.50 ± 0.05 mm	Initial radius where domain walls form
*D*_*w*_	0.100 ± 0.005 mm	Diffusion constant per length expanded
$v_{w}^{iR}$	0.06 ± 0.02	Wall velocity: transverse displacement per length expanded. Obtained by tracking sectors; only observable for strains (*i*) sweeping through mCherry (*R*).
Key Parameters		
$L_{s}^{ij}$	$D_{w}/(v_{w}^{ij}{)}^{2}$	Length expanded when selection dominates diffusion for linear expansions
*κ*_*ij*_	$\sqrt{R_{0}/L_{s}^{ij}}$	Strength of selection versus inflation and diffusion for radial expansions
*ϕ*_*c*_	$\sqrt{8D_{w}/R_{0}}$	Characteristic angular correlation length between strains
Independent Variables		
*R*	–	Radius of the colony
*L*	*R* − *R*_0_	Colony length expanded
*ϕ*	–	Polar coordinate angle

#### Average fractions

The average fraction of strain *i* at a length expanded of *L* = *R* − *R*_0_ is defined as

$$\begin{array}{c} F_{i}\left(L\right)=\frac{1}{2\pi }\int_{0}^{2\pi }d\phi \langle f_{i}\left(\phi ,L\right)\rangle \end{array}$$ (1)

where *f*_*i*_(*ϕ*, *L*) is the local fraction of strain *i* at angle *ϕ* and length *L* (i.e. at a pixel specified in polar coordinates by *ϕ* and *L*). The angular brackets represent an average over many range expansions and *f*_*i*_ is normalized such that ∑_*i*_ *f*_*i*_(*ϕ*, *L*) = 1 for each location in the colony as discussed in the Image Analysis section. In the neutral case, the average fraction of each strain should equal their inoculated fractions and should be independent of length expanded. Selection forces the average fractions of less fit strains to decrease.

**Fig 2:**
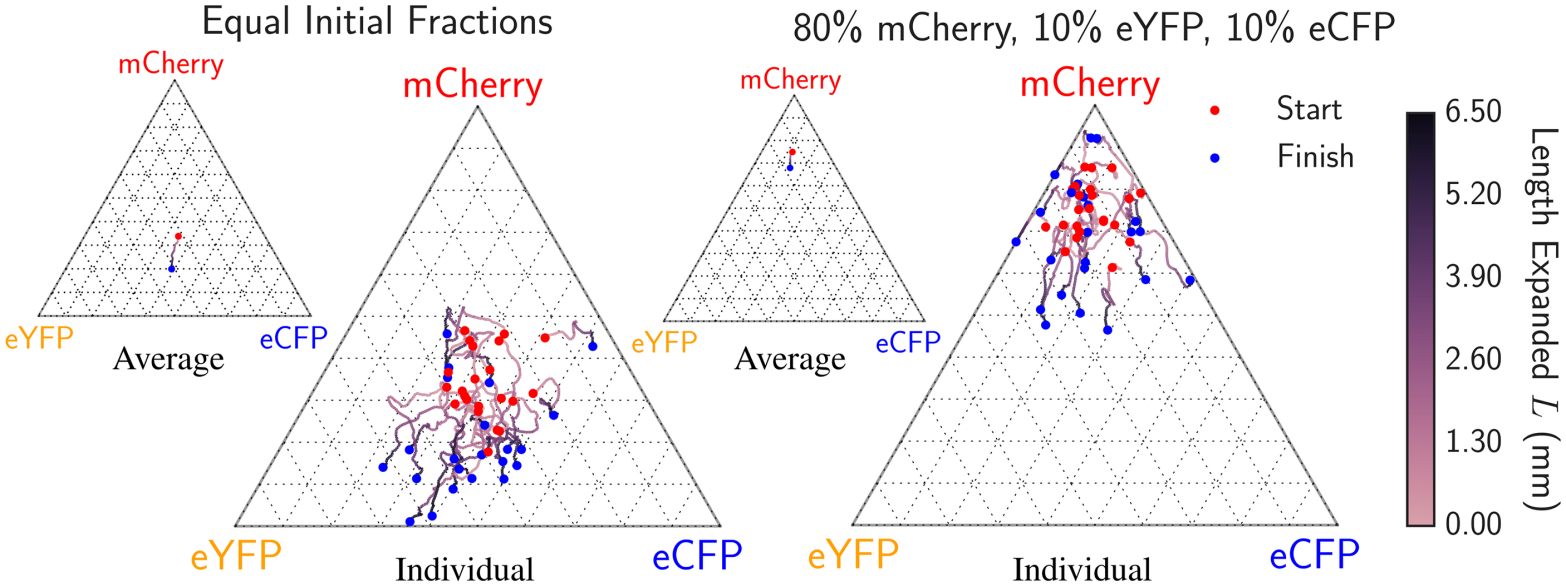
Average fraction of each genotype as a function of length expanded for 20 radial expansions each when equal fractions of eCFP, eYFP, and mCherry were inoculated (left) and when 10% eCFP, 10% eYFP, and 80% mCherry were inoculated (right). The red dot indicates the composition at the radius *R*_0_ = 3.50 mm where distinct domain walls form and the blue dot indicates the composition at the end of the experiment. The red dots are dispersed about the initial inoculated fractions due to the stochastic dynamics at the early stages of the range expansions when *R* < *R*_0_. The highly stochastic trajectories illustrate the importance of genetic drift at the frontier in the *E. coli* range expansions. The smaller ternary diagrams display the average fraction over all expansions vs. length expanded for each set of experiments. For both initial conditions, we see a small systematic drift away from the mCherry vertex indicating that the mCherry strain has a lower fitness, in agreement with the independent radial expansion velocities of each strain (see Table 1). Note that two replicates on the right resulted in the complete extinction of eCFP due to strong spatial diffusion, indicated by the trajectories pinned on the absorbing line connecting the eYFP and mCherry vertices.

We measured the average fraction versus radial length expanded in two separate sets of experiments where we inoculated different fractions of our eYFP, eCFP, and mCherry strains. In one experiment, we inoculated the eYFP, eCFP, and mCherry strains with equal initial fractions of 33% while in the other we inoculated 80% of the mCherry strain and 10% each of the eCFP and eYFP strains. We conducted 20 replicates in each case and calculated the average fraction of each strain using our image analysis package. Fig 2 displays the trajectories of the 20 expansions and the mean trajectory (the average fraction) as ternary composition diagrams for both sets of initial conditions [37].

In both sets of experiments, we observed a systematic drift away from the mCherry vertex as a function of radius as illustrated by the mean trajectories shown as insets. We witnessed two cases where the 10% initial inoculant of the eCFP strain became extinct, represented by the pinning of trajectories to the absorbing boundary connecting the eYFP and mCherry vertex, a consequence of the strong genetic drift at the frontiers of our *E. coli* range expansions. These measurements indicate that the mCherry strain was less fit than the eCFP and eYFP strains, consistent with the order of the radial expansion velocities.

#### Two-point correlation functions

Next, we measured the two-point correlation functions given by

$$\begin{array}{c} F_{ij}\left(\phi ,L\right)=\frac{1}{4\pi }\int_{0}^{2\pi }d\phi^{\prime }\langle f_{i}(\phi^{\prime },L)f_{j}(\phi^{\prime }+\phi ,L)+f_{j}(\phi^{\prime },L)f_{i}(\phi^{\prime }+\phi ,L)\rangle , \end{array}$$ (2)

where *f*_*i*_(*ϕ*, *L*) is again the local fraction of strain *i* at angle *ϕ* and expansion length *L*. *F*_*ij*_ gives us the probability that strain *i* is located at an angular distance of *ϕ* away from strain *j* at a length expanded *L*. Note that *F*_*ij*_ = *F*_*ji*_ and *F*_*ij*_(*ϕ*) = *F*_*ij*_(−*ϕ*). Although the average fraction is constant in the neutral case, the two-point correlation functions broaden due to the coarsening of genetic domains [2]. Neutral *q*-color Voter models analytically predict the form of the two-point correlation functions [2] as seen in equation (S1.3) in S1 Appendix.

Deviations from neutral predictions are caused by selection. Analytical results describing these deviations are not available for reasons discussed in the S1 Appendix (the hierarchy of moments does not close); numerical simulations must be used to calculate the precise shape of the correlation functions as seen in second half of our Results section. Regardless, selection-induced deviations can be understood in the limit of both large and small angular separations. For large angular separations, spatial correlations will be negligible; the two-point correlation functions will consequently factorize and plateau at the value *F*_*ij*_ = *F*_*i*_*F*_*j*_ where *F*_*i*_ is the average fraction at length *L* from above. Therefore, in neutrality, the two-point correlation functions *F*_*ij*_ should plateau at $F_{ij}=F_{i}^{0}F_{j}^{0}$, the product of the initial fractions inoculated of strains *i* and *j* (in neutrality, *F*_*i*_ does not change). Selection can thus be identified by comparing the experimentally measured plateau value to the neutral prediction value. Furthermore, in the limit of zero angular separation, it is known that ∂_*ϕ*_*F*_*ij*_ measures the density of *ij* domain walls [2] (where *i* ≠ *j*). In general, if strain *i* is less fit than the other strains, it will have fewer domain walls, decreasing the domain-wall density and thus the slope near *ϕ* = 0.

**Fig 3:**
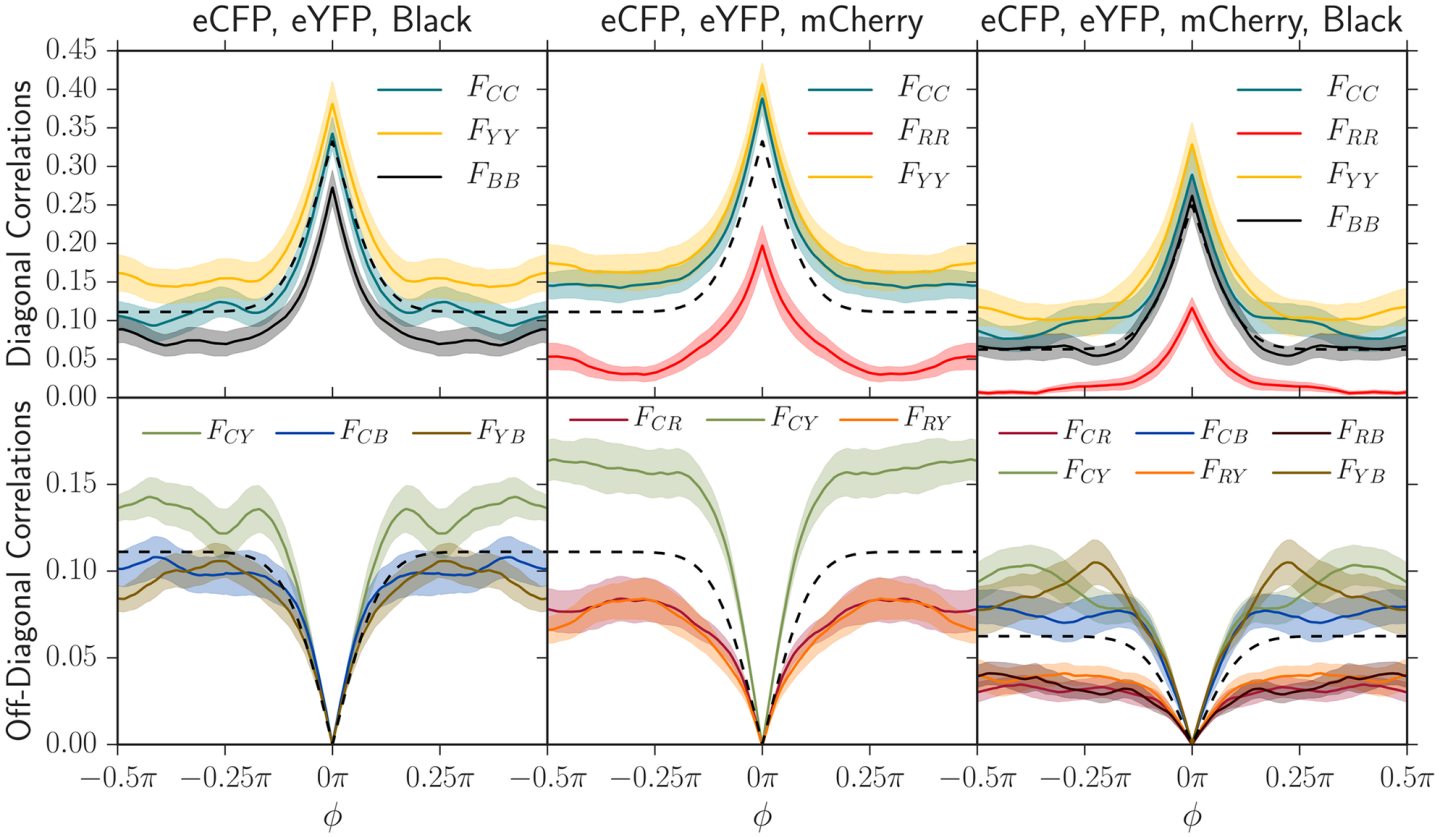
Two-point angular correlation functions *F*_*ij*_(*L*, *ϕ*) at a length expanded of *L* = *R* − *R*_0_ = 6.5 mm (*R* = 10 mm, *R*_0_ = 3.5 mm) in three sets of experiments where we inoculated 20 replicates with equal fractions of our eCFP, eYFP, and black strains (left), then eCFP, eYFP, and mCherry (center), and finally all four strains (right). The shaded regions in these plots indicate standard errors of the mean. Using the measured diffusion coefficient *D*_*w*_ and initial radius where domain walls form *R*_0_ (see Table 2), we also plot the theoretical neutral two-point correlation functions (black dashed line; see eq. (S1.3)). The colors of each plotted correlation function were chosen to correspond to their composite strain colors; for example, two-point correlation correlation functions associated with mCherry were red or were blended with red. The subscripts correspond to the color of each strain: *C* = eCFP, *Y* = eYFP, *R* = mCherry, and *B* = Black. As judged by the magnitude of the deviation from neutral predictions, the black strain has a small selective disadvantage relative to eCFP and eYFP and the mCherry strain has an even greater disadvantage, in agreement with the independent radial expansion velocities of each strain (see Table 1).

We measured the correlation functions between each pair of strains in three sets of experiments where we inoculated equal well-mixed fractions of the eCFP, eYFP, and black strains, then eCFP, eYFP, and mCherry, and then finally all four strains. We conducted 20 replicates of each experiment, measured all two-point correlation functions at the final radius of *R* = 10 mm corresponding to a length expanded of *L* = *R* − *R*_0_ = 6.5 mm, and averaged the results. In Fig 3, we plotted the neutral correlation function prediction and compared it to the experimentally measured correlation functions.

The two-point correlation functions in the experiment between eCFP, eYFP, and the black strains (first column of Fig 3) are consistent with the order of radial expansion velocities (see Table 1). The correlation between the eCFP and eYFP strains plateaued at a higher value than the neutral prediction while the correlation between eCFP and black plateaued at a lower value, indicating that the eCFP and eYFP strains were more fit. The self-correlation for the black strain, *F*_*BB*_, also plateaued at a value below eCFP, eYFP, and the neutral prediction, further indicating that it had a smaller fitness. The self-correlation data was more noisy than the correlation between strains, however; we consistently found that correlations between strains were better at detecting fitness differences than self-correlations.

In contrast, combining eCFP, eYFP, and mCherry in one set of experiments and all four strains in another revealed that mCherry had a larger fitness defect. Correlation functions including mCherry always plateaued at a significantly smaller value than correlation functions excluding it. Furthermore, off-diagonal (bottom-row of Fig 3) correlation functions involving the mCherry strain had a smaller slope at zero angular separation, indicating that less mCherry domain walls were present and that the mCherry strain was less fit than the others. The two-point correlation functions were thus consistent with the black strain having a small selective disadvantage relative to eCFP and eYFP and the mCherry strain having a larger disadvantage relative to all others.

#### Annihilation asymmetry

The last quantity we measured was the relative rate of annihilations and coalescences per domain wall collision; examples of annihilations and coalescences can be seen on the left side of Fig 1. Many theoretical results exist describing the neutral dynamics of annihilations and coalescences and they are summarized in S1 Appendix. To succinctly quantify the difference between the annihilation and coalescence probabilities per wall collision, we define the “annihilation asymmetry” Δ*P*(*L*) = *P*_*A*_(*L*) − *P*_*C*_(*L*) as the difference in probability of obtaining an annihilation versus a coalescence *per collision* at a distance expanded of *L*. If *q* neutral colors are inoculated in equal fractions, it can be shown that

$$\begin{array}{c} \Delta P=\frac{3-q}{q-1}. \end{array}$$ (3)

Note that in neutrality, the annihilation asymmetry Δ*P* is independent of the length expanded *L*; it depends only on the number of strains *q* inoculated in equal fractions. In the presence of selection, however, less fit strains should be squeezed out as the length expanded *L* increases, forcing *q* and thus Δ*P* to change.

To gain insight into the behavior of Δ*P*, for the case of *q* neutral colors in equal proportions, we have lim_*q*→∞_ Δ*P*(*q*) = −1 (only coalescences), Δ*P*(*q* = 3) = 0 (equal numbers of annihilations and coalescences), and Δ*P*(*q* = 2) = 1 (only annihilations). The quantity Δ*P* thus provides a simple way to characterize the annihilation/coalescence difference in a single curve that varies smoothly between −1 and 1 as 2 ≤ *q* < ∞. In S1 Appendix we develop and discuss the case when strains are inoculated in non-equal proportions (see supplementary equations (S1.8)–(S1.10)); in that scenario, it is useful to define a “fractional *q*” by inverting eq (3) to read *q* = (3 + Δ*P*)/(1 + Δ*P*) (i.e. a fractional *q* can be evaluated for a given Δ*P*).

**Fig 4:**
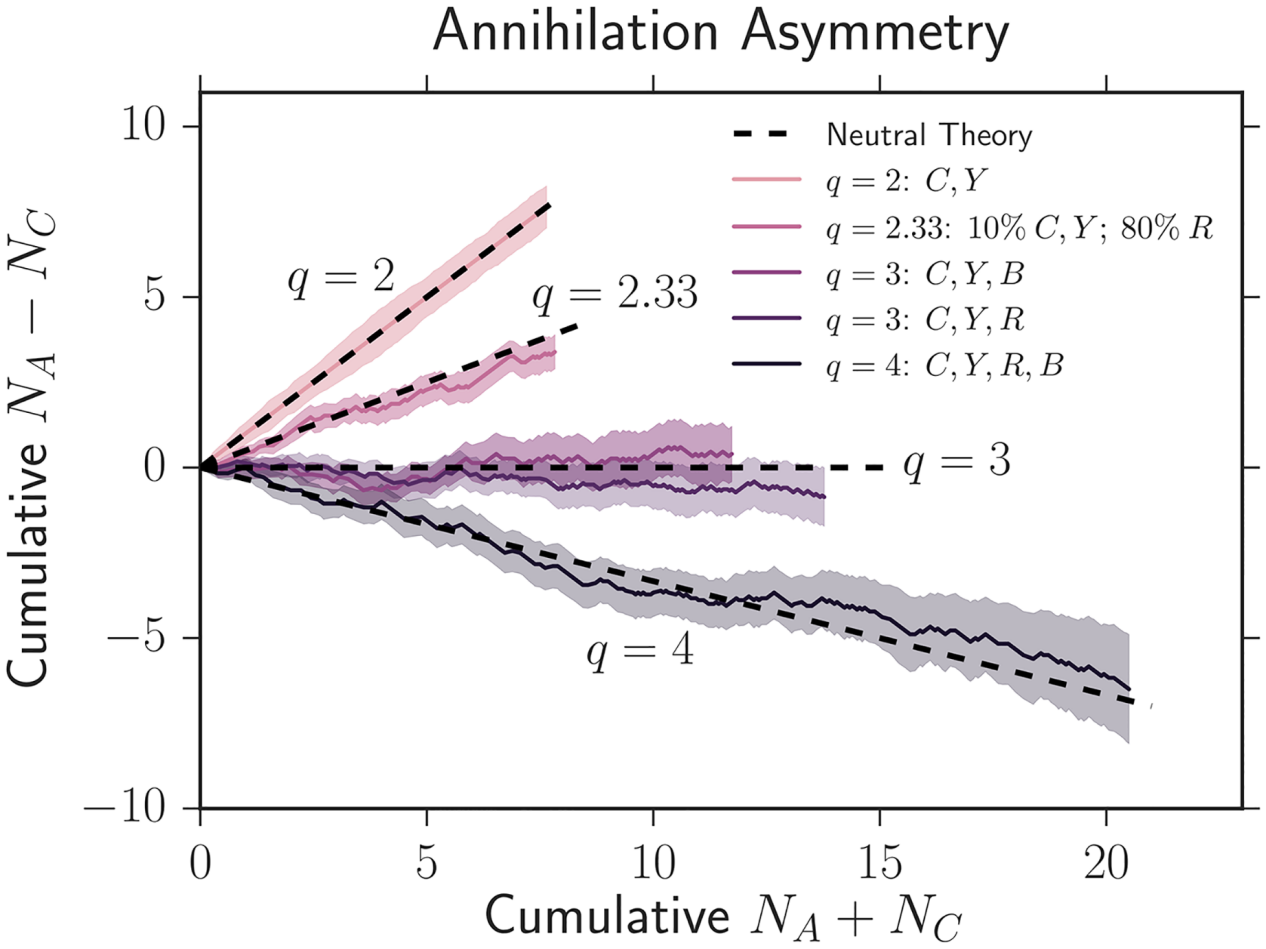
Average cumulative difference in annihilations and coalescences vs. the average cumulative number of domain wall collisions as colonies expand. The slope of this plot gives the annihilation asymmetry Δ*P*. The shaded regions represent the standard error of the mean between many experiments. We use the notation *C* = eCFP, *Y* = eYFP, *B* = black, and *R* = mCherry. Despite the presence of selection, Δ*P* was consistent with the standard neutral theory prediction of eq (3) for *q* = 2, *q* = 3, and *q* = 4 (equal initial fractions of *q* strains), as judged by the overlap of the black dashed lines with the shaded areas in every case. We also explored an initial condition where we inoculated unequal fractions of three strains; we inoculated 10% of both eCFP and eYFP and 80% of mCherry. Our experiments agreed with the prediction of Δ*P* ≈ 0.51, or an effective *q* ≈ 2.33, from the neutral theory developed in supplementary equations (S1.8)–(S1.10).

To experimentally quantify the annihilation asymmetry, we examined the average cumulative difference in annihilations and coalescences vs. the average cumulative number of domain wall collisions as colonies expanded; Δ*P* is given by the slope of this quantity and can be seen in Fig 4 (see Supplementary S1 Fig for a display of cumulative count vs. length expanded). Regardless of which strains were inoculated and their selective differences, our results were consistent with the neutral theory prediction in eq (3) for *q* = 2, *q* = 3, and *q* = 4 as judged by the overlap of the black dashed line with the shaded standard error of the mean in each case. Δ*P* appeared to be constant as a function of length. We also tested an initial condition where we inoculated strains in unequal proportions: we inoculated 10% of eCFP and eYFP and 80% of mCherry. This experiment again matched the neutral prediction of Δ*P* ≈ 0.51 (and correspondingly *q* ≈ 2.33) within error. Evidentally, as discussed in more detail below, certain observables like the average fraction and two-point correlation functions show stronger signatures of selection than others like the annihilation asymmetry.

### Simulation results

In this section, we introduce three key combinations of our random walk model’s input parameters *R*_0_, *D*_*w*_, and $v_{w}^{ij}$ (see Fig 1) that control the evolutionary dynamics of our four competing *E. coli* strains. Using simulation, we show that we can utilize these key combinations to collapse the simulated evolutionary dynamics (focusing on the experimental quantities we measured above: the average fraction, two-point correlation function, and annihilation asymmetry) of an arbitrary number of competing strains in a range expansion.

#### Key parameters

What key combinations of the variables seen on the right side of Fig 1 govern the evolutionary dynamics of our competing strains? Our goal is to describe the dynamics as a function of length expanded by our colonies *L* = *R* − *R*_0_ with *R*_0_ the initial radius where domain walls form, the domain wall diffusion coefficient per length expanded *D*_*w*_ (units of length), and all wall velocities per length expanded $v_{w}^{ij}$ (dimensionless). The two-point correlation functions must include an additional independent variable: the angular distance *ϕ* between strains.

Investigating the width of a single sector of a more fit allele sweeping through a less fit allele, as illustrated on the right of Fig 1, reveals important parameter combinations (see S1 Appendix for additional details). In a linear expansion, the deterministic, selection-induced growth of a sector of genotype *i* sweeping through a less fit genotype *j* will scale as $v_{w}^{ij}L$ while its diffusive growth will scale as $\sqrt{D_{w}L}$. At short lengths expanded, diffusion will thus dominate deterministic growth, and at larger lengths selection will dominate diffusion. A crossover expansion length $L_{s}^{ij}$ [2, 9, 32] beyond which selection dominates follows by equating the deterministic and diffusive growth,

$$\begin{array}{c} \underset{\text{Deterministic}}{\underset{︸}{2v_{w}^{ij}L_{s}^{ij}}}=\underset{\text{Diffusive}}{\underset{︸}{\sqrt{4D_{w}L_{s}^{ij}}}}\;\Rightarrow L_{s}^{ij}=D_{w}/{\left(v_{w}^{ij}\right)}^{2}. \end{array}$$ (4)

The factor of 2 in front of $v_{w}^{ij}$ and 4 in front of *D*_*w*_ arises because we are monitoring the distance between *two* domain walls (i.e. a sector); similar arguments can be applied to describe the motion of individual walls. It is worth noting that the chirality of sector boundaries reported in *E. coli* range expansions [18, 36] would result in a wall velocity pointing in the same direction (left or right) for *every* domain wall. We can ignore this constant bias in our models because sectors will still expand at the same rate despite an additional superposition of all domain walls moving in a specific direction. To avoid complications arising from chirality in this paper, we focus on quantifying the growth of sectors, i.e. the distance *between* two domain walls, as opposed to tracking the motion of an individual domain wall whenever possible. $L_{s}^{ij}$ is the characteristic length that the colony must expand in order for selection to dominate over diffusion for strain *i* sweeping through strain *j* and acts as the first key parameter.

Upon repeating this argument for domains on a radially inflating ring (see S1 Appendix), we identify $L_{I}^{ij}$ [32, 38] as the inflationary analog of $L_{s}^{ij}$: the expansion length beyond which selection dominates over diffusion, and find

$$\begin{array}{c} \underset{\text{Deterministic}}{\underset{︸}{\kappa_{ij}\text{ln}\left(1+\frac{L_{I}^{ij}}{R_{0}}\right)}}=\underset{\text{Diffusive}}{\underset{︸}{\sqrt{1-{\left(1+\frac{L_{I}^{ij}}{R_{0}}\right)}^{-1}}}}. \end{array}$$ (5)

*κ*_*ij*_ is a dimensionless prefactor that can be thought of as an “inflationary selective advantage” controlling the expansion length at which selection dominates over diffusion and is given by

$$\begin{array}{c} \kappa_{ij}=\sqrt{R_{0}/L_{s}^{ij}}=\sqrt{R_{0}{\left(v_{w}^{ij}\right)}^{2}/D_{w}}. \end{array}$$ (6)

**Fig 5:**
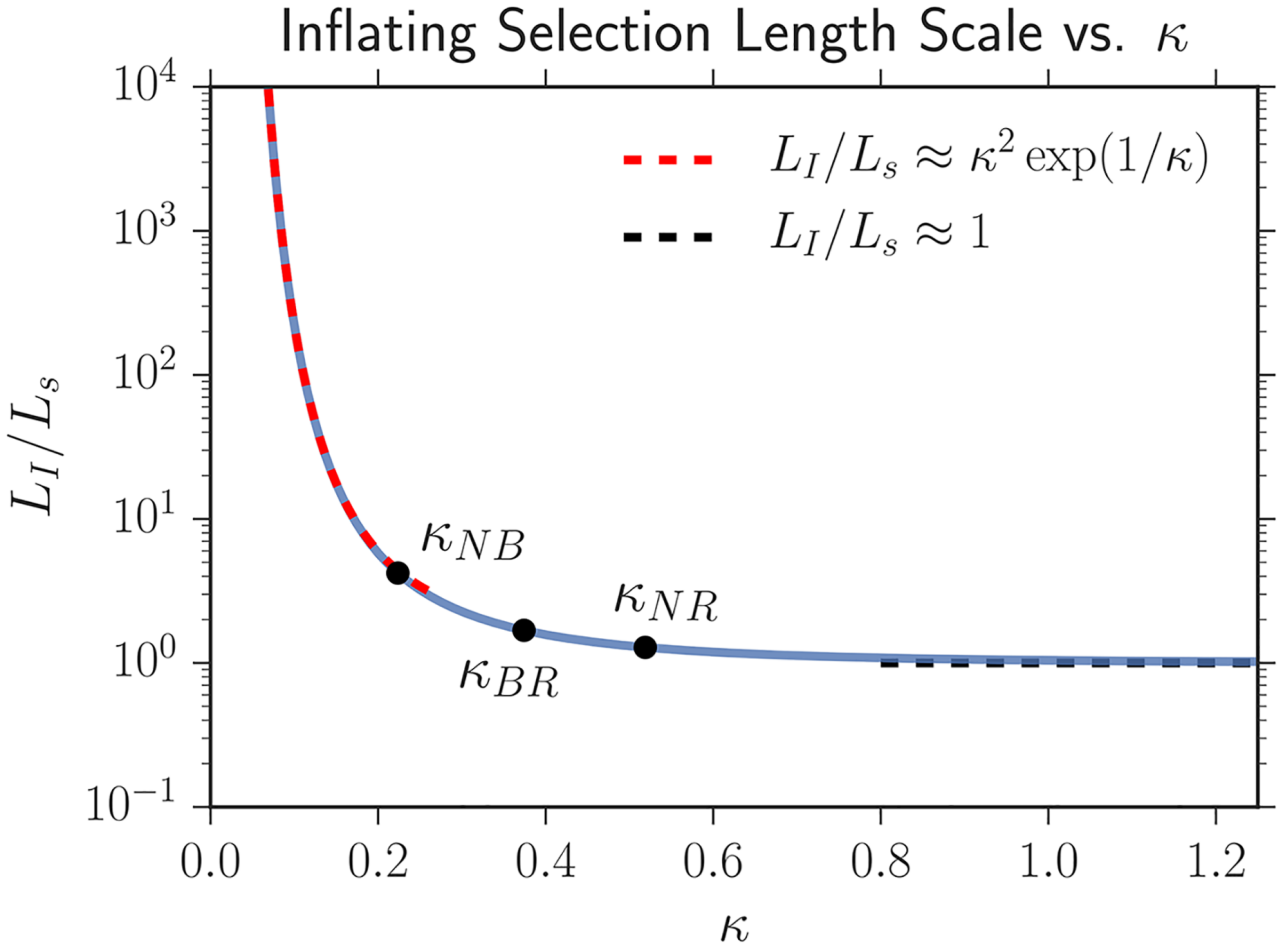
Numerical solution of eq (5) describing how *L*_*I*_ varies as a function of *κ*; analytical asymptotic approximations (eq 7) are overlaid. If *κ* ≳ 1, inflation does not appreciably slow selective sweeps as *L*_*I*_ approaches the linear selection length scale *L*_*s*_. In contrast, if *κ* ≪ 1, the inflationary selection length scale *L*_*I*_ will be many times larger than the linear selection length scale *L*_*s*_, indicating that selection will be weak compared to inflation and diffusion (but will ultimately dominate at very large lengths expanded). The three black points correspond to measurements of the *κ*_*ij*_ that govern the dynamics of our competing strains; *N* stands for the two selectively neutral strains (eCFP and eYFP), *B* for black, and *R* for mCherry (red). See the Predicting experimental results with simulation section for more details.

Fig 5 illustrates the importance of *κ*_*ij*_; it displays the ratio of the inflationary to the linear selection length scale $L_{I}^{ij}/L_{s}^{ij}$ as a function of *κ*_*ij*_ from the numerical solution of eq (5). We find that the ratio of the length scales has the asymptotic behavior

$$\{\begin{array}{ll} \kappa_{ij}\gg 1, & L_{I}^{ij}/L_{s}^{ij}\approx 1\\ \kappa_{ij}\ll 1, & L_{I}^{ij}/L_{s}^{ij}\approx \kappa_{ij}^{2}\;\text{exp}\left(1/\kappa_{ij}\right) \end{array}$$ (7)

Thus, if *κ*_*ij*_ ≫ 1, inflation can be ignored (relative to selection and genetic drift), and the inflating selection length scale approaches the linear selection length scale. In contrast, if *κ*_*ij*_ ≪ 1, the inflationary selection length will be many times larger than the linear selection length scale [32]. As *κ*_*ij*_ becomes smaller, inflation and genetic drift dominate over selection for a larger length expanded. *κ*_*ij*_ is the second key parameter describing the dynamics of our system. Note that in contrast to a linear expansion which just features competition between genetic drift and selection (captured by the quantity $L_{s}^{ij}$), a radial inflation has three, separate, competing effects: genetic drift, selection, and inflation. *κ*_*ij*_ quantifies the strength of selection relative to inflation and diffusion.

The third and final key parameter is the characteristic angular correlation length $\phi_{c}=\sqrt{8D_{w}/R_{0}}$ between selectively neutral genotypes. This parameter arises naturally when analytically calculating the neutral two-point correlation functions from the Voter model (see eq. (S1.3)). The parameter also has an intuitive description. When moving into polar coordinates, the angular diffusion coefficient *D*_*ϕ*_ is related to the standard linear domain wall diffusion coefficient by *D*_*ϕ*_ = *D*_*w*_/*R*^2^. The characteristic scale for the radius is *R*_0_; the angular diffusive growth of domains should consequently scale as $\phi_{c}∼\sqrt{D_{\phi }R_{0}}=\sqrt{D_{w}/R_{0}}$. Note that this characteristic angular length does not depend on the total number of strains; it describes the diffusive coarsening of a single strain sector propagating through one or more other strains.

We have now identified the three key parameters that govern the evolutionary dynamics of our competing strains. $L_{s}^{ij}=D_{w}/(v_{w}^{ij}{)}^{2}$ is the length that a linear expansion must grow in order for selection to dominate over diffusion for strain *i* sweeping through strain *j*, $\kappa_{ij}=\sqrt{R_{0}/L_{s}^{ij}}$ controls whether selection (*κ*_*ij*_ ≪ 1) or inflation (*κ*_*ij*_ ≫ 1) may be neglected relative to other effects in radially inflating expansions, and $\phi_{c}=\sqrt{8D_{w}/R_{0}}$ sets the characteristic angular correlation length between selectively neutral genotypes. These key parameters are listed in Table 2.

#### Collapsing the evolutionary dynamics with the key parameters

We used simulations of annihilating and coalescing random walkers constrained to lie on the edge of an inflating ring with deterministic biases due to selection (see the Simulation methods section for additional details) to investigate the effect of the parameters *R*_0_, *D*_*w*_, and the set of all $v_{w}^{ij}$ on the evolutionary dynamics of our competing strains. As we varied *R*_0_, *D*_*w*_, and $v_{w}^{ij}$, we calculated the average fraction of each strain, the two-point correlation functions between strains, and the relative rate of annihilations and coalescences per domain wall collision (the quantities we measured experimentally). We also investigated the role of the three key combinations of parameters $L_{s}^{ij}$, *κ*_*ij*_, and *ϕ*_*c*_ for both linear and radial expansions.

**Fig 6:**
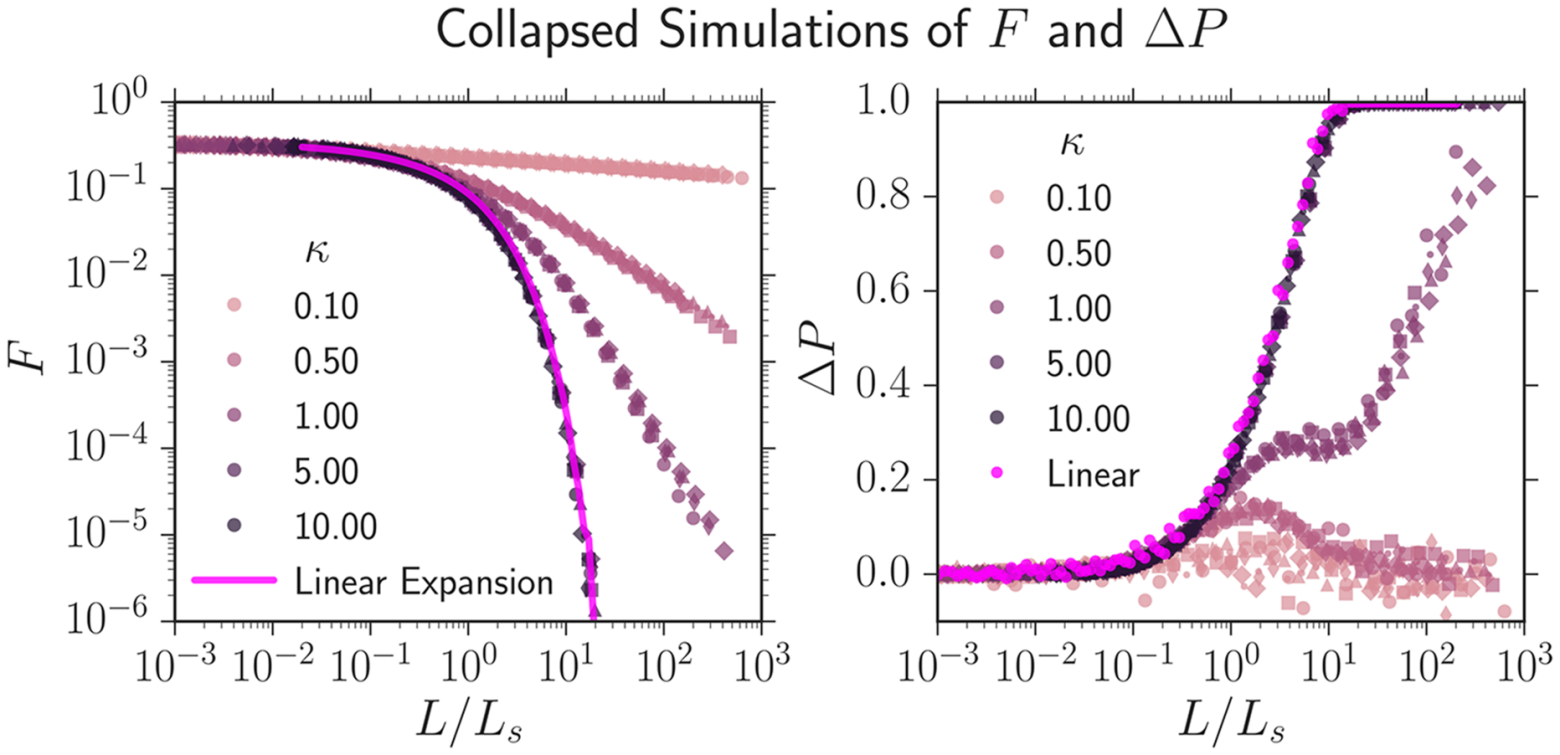
Simulations of the average fraction *F* of a less fit strain and the annihilation asymmetry Δ*P* collapsed onto universal curves parameterized by $\kappa =\sqrt{R_{0}v_{w}^{2}/D_{w}}$. Two neutral strains swept through a less fit strain with a wall velocity *v*_*w*_; each strain was numerically inoculated in equal proportions and the colony’s initial radius was *R*_0_. For identical *κ*, despite different values of *R*_0_ and *v*_*w*_, both *F* and Δ*P* can be collapsed if the length traveled *L* is rescaled by $L_{s}=D_{w}/v_{w}^{2}$, the linear selection length scale. Each universal curve at a fixed *κ* consists of six simulations with different values of *v*_*w*_ and *R*_0_; each set of parameters has a different marker. As *κ* decreases, inflation slows the selective sweep of the more fit strains through the less fit strain as illustrated by the slower decrease of *F*. Δ*P* transitioned from 0 to 1 as the number of strains present in the expansion decreased from *q* = 3 to *q* = 2 (the less fit strain was squeezed out); this is expected from eq (3), Δ*P* = (3 − *q*)/(*q* − 1). Supplementary S3 Fig is identical to this figure except the y-axis of *F*(*L*/*L*_*s*_, *κ*) is placed on a linear scale; this may be useful for comparison with experiments.

We first simulated *q* = 3 competing strains where two neutral strains swept through a third less fit strain with wall velocity *v*_*w*_, similar to our experiments with two neutral strains (eCFP and eYFP) and the less fit mCherry strain. The three strains were numerically inoculated in equal proportions. Note that in this simulation, there was only one non-zero *v*_*w*_ and consequently one $L_{s}=D_{w}/v_{w}^{2}$ and one $\kappa =\sqrt{R_{0}/L_{s}}$. We varied *v*_*w*_ from 10^−3^ ≤ *v*_*w*_ ≤ 10^−1^ and *N*_0_ from 10^2^ ≤ *N*_0_ ≤ 10^5^ (altering *R*_0_ = *N*_0_*a*/(2*π*)) and computed the average fraction *F* of the less fit strain and the annihilation asymmetry Δ*P*. We found that both *F* and Δ*P* from simulations with identical *κ*, despite different values of *R*_0_ and *v*_*w*_, collapsed if *L*, the length traveled was rescaled by *L*_*s*_ as seen in Fig 6. Each curve in Fig 6 consists of six collapsed simulations with unique values of *R*_0_ and *v*_*w*_ but with the same value of *κ*. Further simulations revealed that the two-point correlation functions *F*_*ij*_ could be collapsed from simulations with identical *κ* if *L* was rescaled by *L*_*s*_ and *ϕ* was rescaled by $\phi_{c}=\sqrt{8D_{w}/R_{0}}$ provided *ϕ*_*c*_ ≪ 2*π* (see Supplementary S2 Fig).

We now consider the collapsed curves *F*(*L*/*L*_*s*_, *κ*) and Δ*P*(*L*/*L*_*s*_, *κ*) as a function of the parameter *κ* as seen in Fig 6. *κ* had a pronounced effect on both quantities. For *κ* ≳ 5 the dynamics of *F* and Δ*P* approached the dynamics of a linear expansion at all *L*/*L*_*s*_, illustrated by the bright pink line on the left and the bright pink dots on the right of Fig 6; the more fit strain swept so quickly through the less fit strain that the colony’s radial expansion could be ignored. As *κ* decreased, the less fit strain was squeezed out more slowly due to the inflation of the frontier, resulting in slower transitions from *q* = 3 to *q* = 2 colors and consequently slower transitions from Δ*P* = 0 to Δ*P* = 1. For *κ* ≪ 1, Δ*P* barely shifted from 0 over the course of the simulation. Interestingly, Δ*P* peaked at a finite *L*/*L*_*s*_ for small *κ*; it is not clear what causes this effect, but it may be related to the transition from linear to inflation-dominated dynamics as *L* increases.

Additional simulations revealed that for expansions composed of many strains with different fitnesses (multiple $v_{w}^{ij}$) and consequently various *κ*_*ij*_, all of our observables (*F*, Δ*P*, and *F*_*ij*_) could again be collapsed onto a master curve by rescaling *L* by any one of the selection length scales (i.e. $L/L_{s}^{ij}$) and by rescaling *ϕ* by *ϕ*_*c*_; the set of *κ*_*ij*_ specified the master curve. An example of a simulation with that exhibits collapsed dynamics for three *κ*_*ij*_ can be seen in Supplementary S4 Fig.

To summarize the results of this section, we found that we could collapse the average fraction *F*, annihilation asymmetry Δ*P*, and the two-point correlation functions *F*_*ij*_ by

$$F(L,D_{w},R_{0},\{v_{w}^{ij}\})\to F(L/L_{s}^{ij},\{\kappa_{ij}\})$$ (8)

$$\Delta P(L,D_{w},R_{0},\{v_{w}^{ij}\})\to \Delta P(L/L_{s}^{ij},\{\kappa_{ij}\})$$ (9)

$$F_{ij}(\phi ,L,D_{w},R_{0},\{v_{w}^{ij}\})\to F_{ij}(\phi /\phi_{c},L/L_{s}^{ij},\{\kappa_{ij}\})$$ (10)

where the brackets indicate a *set* of variables parameterized by *i* and *j* (i.e. $\left\{v_{w}^{ij}\right\}$ represents the set of all *ij* wall velocities). As long as *L* was rescaled by any selection length scale $L_{s}^{ij}$ and *ϕ* was rescaled by the characteristic angular correlation length *ϕ*_*c*_, the set of {*κ*_*ij*_} completely dictated the evolutionary dynamics.

### Predicting experimental results with simulations

A major goal of this paper is to test if the annihilating and coalescing random-walk model can predict the experimental evolutionary dynamics of our four competing strains (alleles) with different fitnesses (radial expansion velocities). To the best of our knowledge, analytical results for the random-walk model are unavailable (as discussed in S1 Appendix); we consequently used our simulations to predict the dynamics. In this section we quantify the three key parameter combinations for our *experimental* expansions and then use them to predict the evolutionary dynamics of all four of our competing *E. coli* strains in an independent experiment.

In the last section, we found that our simulation dynamics could be collapsed onto master curves for a fixed set of $\kappa_{ij}=\sqrt{R_{0}/L_{s}^{ij}}$ by rescaling the length expanded *L* by any single $L_{s}^{ij}=D_{w}/(v_{w}^{ij}{)}^{2}$ and by rescaling *ϕ* by $\phi_{c}=\sqrt{8D_{w}/R_{0}}$. These simulated master curves were invariant to the alteration of simulation parameters provided that the set of *κ*_*ij*_ remained the same. This insight allowed us to develop a novel method of characterizing the *experimental* dynamics. Namely, we could *experimentally* determine $L_{s}^{ij}$, *κ*_*ij*_, and *ϕ*_*c*_, collapse the experimental data the same way as the simulations (i.e. $L/L_{s}^{ij}$, *ϕ*/*ϕ*_*c*_), and compare the two to predict the dynamics of many competing alleles in a range expansion. As discussed below, this technique ultimately allowed for accurate predictions of the evolutionary dynamics of the four competing strains and, surprisingly, allowed us to make much more precise measurements of selective differences between strains.

As mentioned above in the Experimental Results section, using the procedures outlined in the Materials and Methods, we had previously determined *R*_0_ = 3.50 ± 0.05 mm and *D_w_* = 0.100 ± 0.005 mm (Table 2). In order to fit $L_{s}^{ij}=D_{w}/(v_{w}^{ij}{)}^{2}$ and $\kappa_{ij}=\sqrt{R_{0}/L_{s}^{ij}}$, however, we needed to measure $v_{w}^{ij}$. By tracking the growth of a more fit sector sweeping through a less fit strain (see the Materials and methods), we found that each strain swept through mCherry with a wall velocity of $v_{w}^{iR}=0.06\pm 0.02$ (as seen in Table 2); we could not detect the wall velocity of the eYFP and eCFP sweeping through the black strain.

In principle, the measured values of *R*_0_, *D*_*w*_, and $v_{w}^{ij}$ should have allowed us to totally calibrate the three key parameter combinations. For example, $\phi_{c}=\sqrt{8D_{w}/R_{0}}=0.48\pm 0.01$. The value of $\kappa_{ij}=\sqrt{R_{0}/L_{s}^{ij}}$ followed from the measurement of $L_{s}^{ij}$ using the known value of *R*_0_. Unfortunately, the final parameter $L_{s}^{ij}=D_{w}/(v_{w}^{ij}{)}^{2}$ was more difficult to calibrate. Using $v_{w}^{iR}=0.06\pm 0.02$, we found that $L_{s}^{iR}=30\pm 20\;mm$; the error on this value was too large for it to be predictive in our simulations. Furthermore, as we were unable to accurately measure the wall velocity of the eCFP/eYFP strains sweeping through the black strain, we could not calculate the corresponding selection length scale. We therefore needed a new technique to determine $L_{s}^{ij}$. As our eCFP and eYFP strains were neutral within error, we treated our system as composed of one neutral (*N*) eCFP/eYFP strain, a red (*R*) mCherry strain, and a black (*B*) strain (*q* = 3 colors). As the eCFP/eYFP expanded faster than the black followed by the mCherry strain, we needed to determine the values of $L_{s}^{NR}$, $L_{s}^{NB}$, and $L_{s}^{BR}$.

**Fig 7:**
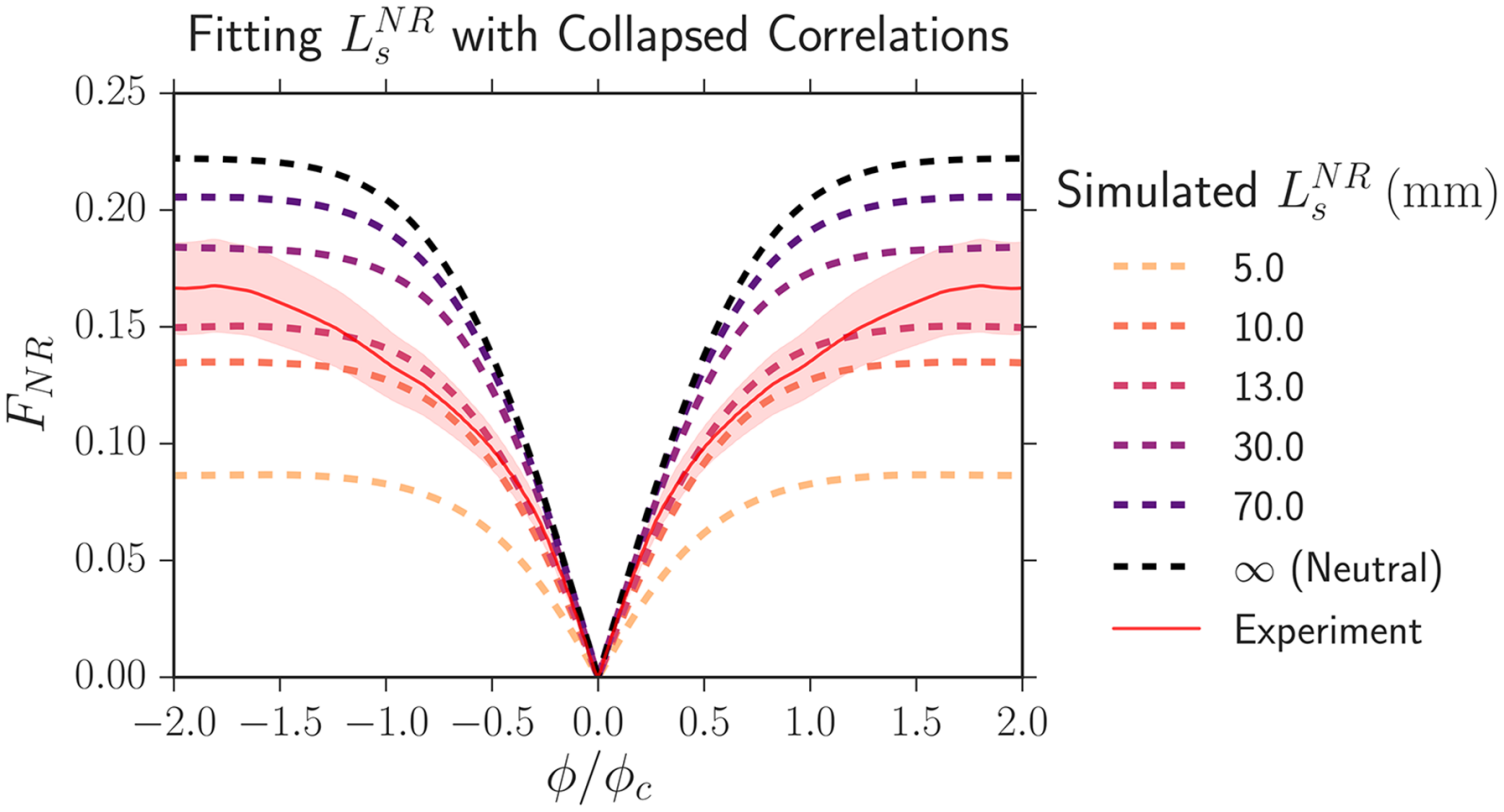
The collapsed two-point correlation function *F*_*NR*_ between our eCFP/eYFP strain (*N*) and our mCherry strain (*R*), which were inoculated at fractions of 2/3 and 1/3, respectively, at a length expanded of *L* = 6.5 mm. The solid red line is experimental data and the shaded region is its standard error of the mean. The dashed lines are the simulated universal correlation functions corresponding to different values of $L_{s}^{NR}$. The best fitting selection length scale is $L_{s}^{NR}=13\pm 3\;\text{mm}$.

To fit $L_{s}^{ij}$ more precisely than that from our direct measurement of wall velocity, we competed pairwise combinations of strains in range expansions (i.e. the eCFP/eYFP strain vs. mCherry) and calculated the two-point correlation functions *F*_*ij*_(*L*, *ϕ*) at the maximum length expanded of *L* = 6.5 mm. As there were only two competing strains, there was only one *L*_*s*_. To fit the value of *L*_*s*_, we began by rescaling the experimental length expanded *L* by *L*_*s*_ and *ϕ* by *ϕ*_*c*_ (Table 2) and calculated the resulting $\kappa =\sqrt{R_{0}/L_{s}}$. Note that *L*_*s*_ *simultaneously* rescales the length expanded *L* by *L*_*s*_ and sets the value of $\kappa =\sqrt{R_{0}/L_{s}}$, changing the shape of the collapsed correlation function. We then ran a simulation at the set value of *κ* (the chosen simulated values of *L*_*s*_ and *ϕ*_*c*_ did not matter due to the collapse) and then compared the collapsed experimental dynamics to our simulation. Fig 7 illustrates the fitting procedure by displaying the experimentally rescaled two-point correlation function *F*_*NR*_ (the solid red line) at a length expanded of *L* = 6.5 mm between our eCFP/eYFP strain (*N*) and our mCherry strain (*R*) (inoculated at fractions of 2/3 and 1/3 respectively) and simulated universal correlation functions corresponding to different values of *L*_*s*_ (dashed lines).

To determine the best-fitting value of *L*_*s*_, we calculated the sum of the squared displacements weighted by the inverse of the experimental standard error squared between experiment and simulation. The best-fitting *L*_*s*_ was determined by finding the value which minimized the weighted sum of squares. To estimate the error in our fit, we assigned each potential value of *L*_*s*_ a probability proportional to the inverse of the weighted sum of squares, normalized the probability distribution, and set the error in our fit of *L*_*s*_ to the confidence intervals of the probability distribution.

Our fit values of $L_{s}^{ij}$ and *κ*_*ij*_ using this technique are listed in Table 3; the values of *κ*_*ij*_ are also plotted in Fig 5. Although this technique was about a factor of 5 more precise than using the measured wall velocities $v_{w}^{iR}$ to determine $L_{s}^{ij}$, the upper bounds of the 95% confidence intervals were still very large as seen in Table 3; the potential values of *L*_*s*_ had a very large tail.

**Table 3:** Results of fitting $L_{s}^{ij}$ from the two-point correlation functions *F*_*ij*_ and the resulting $\kappa_{ij}=\sqrt{R_{0}/L_{s}^{ij}}$ using the two-point correlation function technique. “CI” stands for confidence interval.

Strain *i*	Strain *j*	$L_{s}^{ij}\;\left(mm\right)$	95% CI (mm)	$\kappa_{ij}=\sqrt{R_{0}/L_{s}^{ij}}$
eYFP/eCFP	mCherry	13 ± 3	[7, 38]	0.52 ± 0.07
Black	mCherry	25 ± 7	[14, 250]	0.37 ± 0.06
eYFP/eCFP	Black	70 ± 20	[30, 650]	0.22 ± 0.04

**Fig 8:**
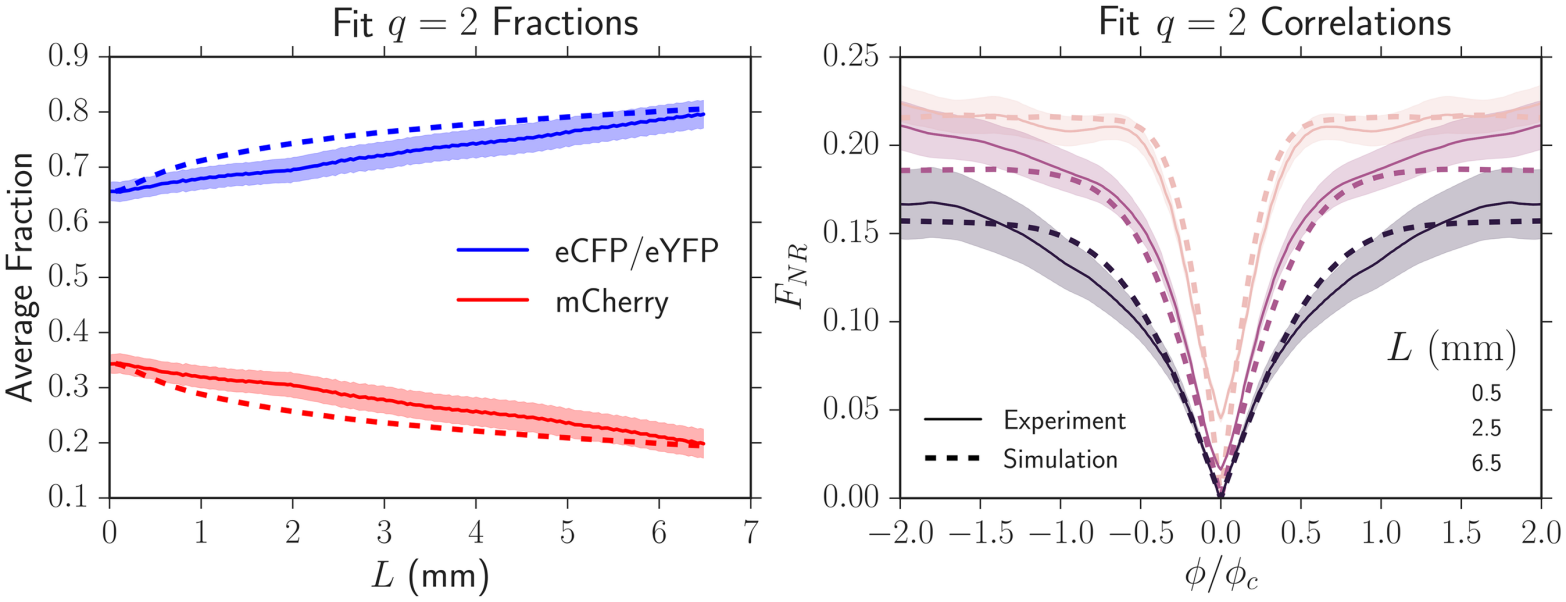
Experimental average fractions and two-point correlation functions (solid lines) and their predicted dynamics (dashed lines) by using the fit *L*_*s*_ from Fig 7. The shaded region is the standard error of the mean. The simulated dynamics closely match the experimental dynamics, suggesting that our fitting technique to extract *L*_*s*_ is robust and can be used to describe the dynamics of our strains at all *L*.

To test that the resulting *L*_*s*_ and *κ* could accurately predict the experimental dynamics at all *L* and not just the *L* where the correlation functions were fit, we plotted the experimental average fraction and correlation functions (solid lines, Fig 8) as we varied *L* and compared their values to those predicted by simulation (dashed lines, Fig 8). Fig 8 uses the same set of experimental data as that from Fig 7. The simulation using the fit parameters always closely tracked the experimental values at all *L*, suggesting that our fitting technique was robust and could be used to describe the dynamics of our strains.

**Fig 9:**
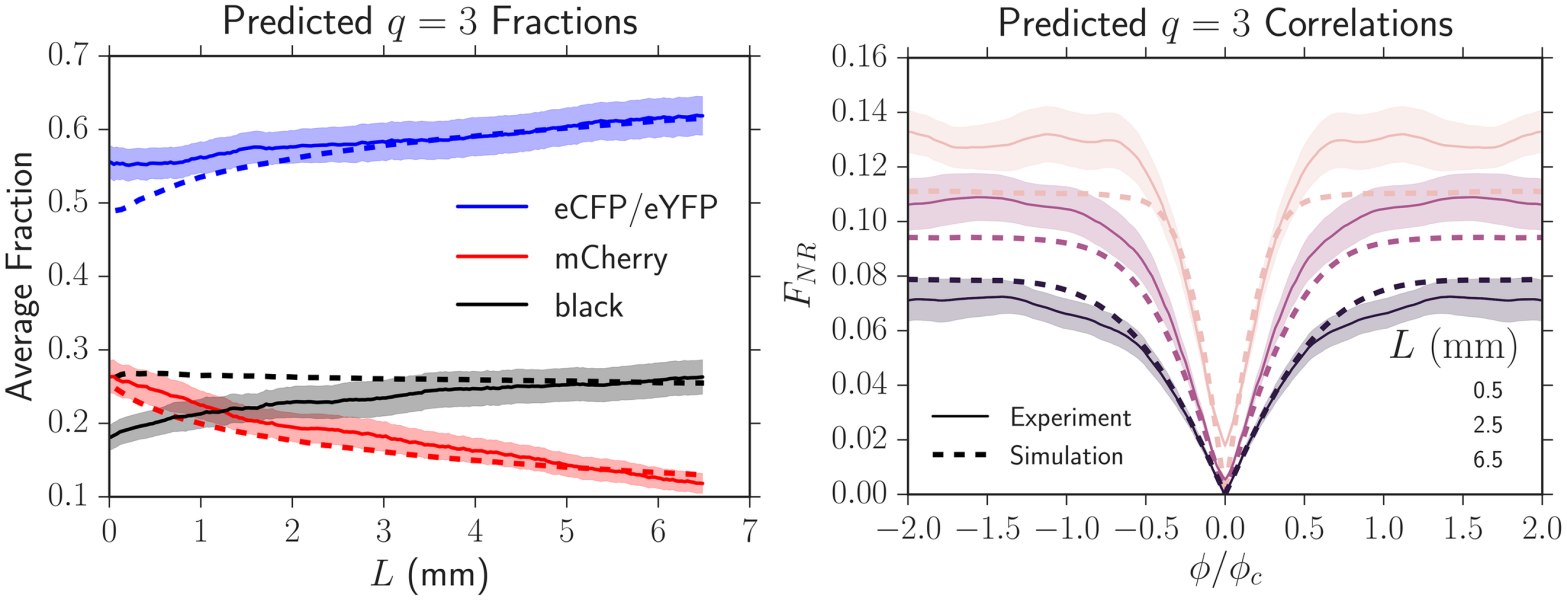
Experimental average fractions and two-point correlation functions (solid lines) of the four strains grown together with equal initial proportions and their predicted dynamics (dashed lines) from simulations using the set of $L_{s}^{ij}$, *κ*_*ij*_, and *ϕ*_*c*_ measured in independent experiments. No additional fitting parameters were used. The shaded region is the standard error of the mean. The simulated dynamics closely matched the experimental dynamics except at small lengths expanded (*L* ≲ 3 mm) where the black strain introduced significant image analysis artifacts (see Supplementary S5 Fig).

Having determined $L_{s}^{ij}$ and *κ*_*ij*_ from pairwise competitions between strains, we tested if we could predict the average fraction, the two-point correlation functions, and the annihilation asymmetry when the four *E. coli* strains were grown together (treating the eYFP and eCFP strains as neutral, so *q* = 3) in an independent experiment. We inoculated the four strains in equal proportions. Fig 9 shows experimental measurements of the average fractions and two-point correlation functions (solid lines) together with simulated predictions (dashed lines) that used the independently fit values; no additional fitting parameters were used. The predicted average fractions and correlation functions closely tracked the dynamics for *L* ≳ 3 mm. We attribute the deviations for *L* ≲ 3 mm to image analysis artifacts resulting from the presence of the black strain (see the Image Analysis section in the Materials and methods). At the largest length expanded of *L* = 6.5 mm where artifacts were minimal, the experiments matched the predictions within error. All average correlation functions at this length expanded were successfully predicted by the simulations; we only display *F*_*NR*_ for simplicity.

In addition to predicting the average fractions and correlation functions, the simulation with our fit $L_{s}^{ij}$ and *κ*_*ij*_ predicted that the annihilation asymmetry would deviate only slightly from neutrality (at most a change of 0.1) over the length expanded by our colonies in every experiment, agreeing with our findings (Fig 4). This can be readily observed in Fig 6 which displays a simulation of two neutral strains (our eCFP and eYFP strains) and a less fit strain (our mCherry strain) inoculated in equal fractions. If we rescale the maximum distance expanded by our colonies of *L*_max_ = 6.5 mm by the smallest selection length (this results in the largest possible change of Δ*P*) of $L_{s}^{NR}=13\;\text{mm}$, *L*_max_/*L*_*s*_ ∼ 0.5 and Δ*P* increases from 0 (neutrality) to at most 0.1. This small deviation from neutrality is within the uncertainty of our experimental measurement of Δ*P*. Evidently, certain quantities, like the average fraction and correlation functions, show signs of selection before others (in this case, the annihilation asymmetry).

The quantitative agreement between our model and our experiments suggests that the one-dimensional annihilating-coalescing random walk model can indeed be used to predict the dynamics of many competing strains with different fitnesses in a range expansion.

## Discussion

We investigated the evolutionary dynamics of four competing strains of *E. coli* in radial range expansions with differing selective advantages. We measured the average fraction *F*_*i*_ of each strain, the two-point correlation functions *F*_*ij*_ between strains, and the annihilation asymmetry Δ*P* with our image analysis toolkit [34]. Our simulations, which model the expansions as a one-dimensional line of random walkers subject to deterministic drift on an inflating ring, showed us that these three quantities could be collapsed onto universal curves for a fixed set $\kappa_{ij}=\sqrt{R_{0}/L_{s}^{ij}}$ when the length expanded by the colony *L* was rescaled by any of the lengths $L_{s}^{ij}=D_{w}/(v_{w}^{ij}{)}^{2}$ and the angular distance between strains *ϕ* was rescaled by $\phi_{c}=\sqrt{8D_{w}/R_{0}}$.

To test if the random walk model could predict experimental dynamics, we independently calculated experimental values of $L_{s}^{ij}$, *κ*_*ij*_, and *ϕ*_*c*_ and compared the dynamics between the two. The simulations accurately predicted the dynamics of the average fraction, correlation functions, and annihilation asymmetry when all four of our strains were present with no additional fitting parameters. The annihilation asymmetry Δ*P* is a quantity unique to range expansions with three or more strains and, to the best of our knowledge, has not been studied previously. Our results illustrate the importance of considering domain wall annihilation and coalescence when more than two strains are present and suggest that the annihilating-coalescing random-walk model can act as a useful predictive tool when describing the evolutionary dynamics of range expansions composed of an arbitrary number of competing alleles with different fitnesses.

Along the way, we introduced a new technique that compared universal simulated correlation functions to experimental correlations to fit $L_{s}^{ij}$. The resulting values of $L_{s}^{ij}$ were about a factor of 5 more precise than directly evaluating $L_{s}^{ij}=D_{w}/(v_{w}^{ij}{)}^{2}$ with the wall velocities extracted from the growth of sectors. Given our fit $L_{s}^{ij}$, we evaluated $v_{w}^{ij}$ using $L_{s}^{ij}=D_{w}/(v_{w}^{ij}{)}^{2}$ and the known *D*_*w*_; we compare these values to those extracted from single sectors in Table 4. The wall velocities from both measurements agreed within error, but the wall velocities obtained from our correlation method were at least a factor of two more precise than tracking single sectors. Although the correlation technique dramatically increased the precision in our evaluation of $L_{s}^{ij}$, the resulting precision increase for $v_{w}^{ij}$ was less pronounced as $v_{w}^{ij}\propto 1/\sqrt{L_{s}^{ij}}$. Nevertheless, it is clear that the correlation technique can be used to precisely extract small differences in fitness between spatially competing strains.

**Table 4:** Quantifying the wall velocities $v_{w}^{ij}$ from our fits of $L_{s}^{ij}$ by using $L_{s}^{ij}=D_{w}/(v_{w}^{ij}{)}^{2}$ and an independently measured *D*_*w*_ which was constant between experiments. The uncertainty in $v_{w}^{ij}$ from the fit $L_{s}^{ij}$ was calculated via error propagation.

Strain *i*	Strain *j*	$L_{s}^{ij}\;\left(mm\right)$: fitting *F*_*ij*_	$v_{w}^{ij}$: from fit $L_{s}^{ij}$	$v_{w}^{ij}$: tracking sectors
eYFP/eCFP	mCherry	13 ± 3	(9 ± 1) × 10^−2^	(6 ± 2) × 10^−2^
Black	mCherry	25 ± 7	(6 ± 1) × 10^−2^	(6 ± 2) × 10^−2^
eYFP/eCFP	Black	70 ± 20	(3.8 ± 0.7) × 10^−2^	Undetectable

Our work illustrates that the annihilating-coalescing random walk model can predict the experimental dynamics of an arbitrary number of competing alleles with different fitnesses in microbial range expansions. It is possible that this model could predict the dynamics of range expansions occurring outside of the laboratory, especially if the expanding organisms’ underlying motion did not completely smear out the population’s spatial structure; the organismal motion could potentially be accounted for by increasing the domain wall diffusion coefficient *D*_*w*_. To predict the dynamics of expansions, however, the annihilating-coalescing walk model relies on a key set of parameters: the set of $L_{s}^{ij}$, the set of *κ*_*ij*_, and *ϕ*_*c*_. We found that the set of $L_{s}^{ij}$ could *not* be predicted from the independent radial expansion velocities of our strains; standard techniques [17] using the relative ratio of expansion velocities to predict $v_{w}^{ij}$, and thus $L_{s}^{ij}$, yielded inconsistent results (see S2 Appendix where we quantify the discrepancy and postulate why it occurred). As the set of $L_{s}^{ij}$ is so fundamental to the evolutionary dynamics of range expansions, future work should investigate why relative radial expansion velocities could not be used to accurately predict $v_{w}^{ij}$ and thus $L_{s}^{ij}$ and whether this phenomenon is specific to *E. coli* range expansions or our specific strains. It would also be interesting to incorporate the reported super-diffusive motion of domain walls [8, 9] into our simplified simulations and theoretical analysis. The random walk model’s ability to successfully predict the evolutionary dynamics of our experiments suggests that annihilating and coalescing genetic domain walls subject to diffusion and selection-induced displacement provide a useful conceptual framework from which to understand range expansion dynamics.

## Materials and methods

### Strains

We used four *E. coli* strains (labelled BW001, BW002, BW003, and BW012) with a DH5*α* background and plasmids whose sequences coded for spectrally distinguishable fluorescent proteins. The unique colors were obtained by using the plasmid vector pTrc99a [39] and the open reading frame for the respective fluorescent proteins. Strains BW001, BW002, and BW003 expressed eCFP (cyan/blue), Venus YFP (yellow), and mCherry (red) respectively, and were identical to the *E. coli* strains eWM282, eWM284, and eWM40 used in Ref. [40]. Note that these three strains were isogenic and differed *only* by the open reading frames corresponding to their respective fluorescent proteins. The final strain, BW012, was a mutated descendant of strain BW002 (yellow) that fluoresced at a decreased intensity, appearing black, while retaining its ampicillin resistance from the pTrc99a vector. Throughout this work, no additional mutations were introduced or observed. We therefore consider that these four strains correspond to four different alleles. Throughout the paper, we refer to the strains as eCFP, eYFP, mCherry, and black.

### Experimental setup

To prepare saturated cultures, strains were inoculated in 10mL of 2xYT media and were shaken for approximately 16 hours at 37°C. After vortexing each saturated culture and obtaining their concentration via optical density (OD-600) measurements, appropriate volumes (e.g., 1:1:1 mixtures of three strains) were added to an Eppendorf tube with a final volume of 1mL. The Eppendorf tube was then vortexed to uniformly mix the strains. A volume of 2 *μ*L was taken from the vortexed tube and placed on center of a 100 mm diameter Petri dish containing 35 mL of lysogeny broth (LB), ampicillin at a concentration of 100 *μ*g/mL, and 1.25% w/v bacto-agar. The carrier fluid in the resulting circular drop evaporated within 2-3 minutes, depositing a circular “homeland” of well-mixed bacteria onto the plate.

After inoculation, plates were stored for 8 days upside down (to avoid condensation) in a Rubbermaid 7J77 box at 37°C with a beaker filled with water; the water acted as a humidifier and prevented the plates from drying out. The plates were occasionally removed from the box and imaged (at roughly 24 hour intervals) using the brightfield channel to determine the radius of the colony as a function of time. On the eighth day, the plates were imaged in both fluorescent and brightfield channels. The number of replicate plates used are stated next to the respective experimental results. If we noticed that a mutation had occurred during an expansion (mutations usually presented themselves as unexpected large bulges at the front of a colony or as distortions in fluorescent intensity), we discounted the colony.

### Image acquisition and analysis

**Fig 10:**
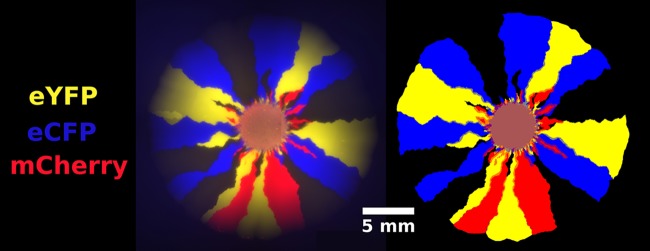
A four-color *E. coli* range expansion (left) and the superimposed binary masks of each channel (right) [34]. Images were acquired for four overlapping quadrants and stitched together to obtain a single image with a large field of view. Overlapping regions were blended to minimize inhomogeneities. To obtain the binary masks, pixels with fluorescence above background noise were marked as “on.” A visual comparison of the raw data and the masks confirm that our binary masks accurately reflect the location and shape of individual sectors.

We imaged our range expansions with a Zeiss SteREO Lumar.V12 stereoscope in four channels: eCFP, eYFP, mCherry (fluorescent channels), and brightfield. In order to analyze a colony with a maximum radius of approximately 10 mm using a single image, we stitched four images together with an overlap of 20% using AxioVision 4.8.2, the software accompanying the microscope. We blended the overlapping areas of the images to lessen the impact of background inhomogeneities. An example of a stitched image can be seen on the left side of Fig 10. Stitching introduced small artifacts such as vertical lines near the center of our expansions; we verified that these did not affect our results.

To extract the local fraction of each strain per pixel, we first created binary masks for each fluorescence channel indicating if the corresponding *E. coli* strain was present. We utilized the “Enhance Local Contrast” (CLAHE) algorithm [41] in Fiji [42], an open-source image analysis platform, to help correct for inhomogeneities in background illumination. After applying the CLAHE algorithm, a combination of automatic thresholding and manual tracing yielded a binary mask of each channel, an example of which is shown in Fig 10; the image on the left is an overlay of an experimental range expansion’s fluorescent channels and the image on the right is the overlay of the corresponding binary masks. A small amount of manual tracing was required near the edges of our colonies because our fluorescent lamp provided uneven illumination; resulting dark regions could barely be identified above background noise. As we mainly used manual tracing near the edge of the colonies where the monoclonal sectors were well defined, we found that our procedure was very reproducible. To alleviate this problem, future work could utilize brighter strains or a more advanced imaging setup.

We mapped the binary images to the local fraction of each *E. coli* strain in the following way: if *N* binary masks (corresponding to *N* colors) were “on” at a pixel, the local fraction of their corresponding channels was assigned to be 1/*N*. Although this assignment produces inaccuracies (i.e., if one strain occupied 90% of a pixel and the other occupied 10%, our algorithm would register both as 50%), domain boundaries were the only areas besides the homeland and the early stages of the range expansions where multiple strains were colocalized. The black strain was defined to be present at pixels reached by the range expansion in which no other strains were present. Although this definition introduced errors at radii close to the homeland with significant color overlap, the error became negligible at large radii as quantified in Supplementary S5 Fig. Once we determined the fraction of each strain at each pixel, we were able to extract quantities such as the total fraction of each strain in the colony and spatial correlations between strains at a given expansion radius.

The mask in Fig 10 highlights that sector boundaries can be used to determine local strain abundance. Although it is possible to extract the position of every domain wall from each strains’ local fraction, it is challenging to actually *track* a single wall due to collisions between walls. To address this problem, we created a binary mask of the edges in our images and labelled the edges of each domain. Annihilations and coalescences were counted manually within Fiji [42]; automated measures were not accurate enough.

It is worth pointing out that in this paper, we ignore the three-dimensional structure of our colonies and describe them by our two-dimensional images taken with the stereoscope. We justify this approximation because the initial diameter of our colonies is at least a factor of 10 larger than their height (less than 1 mm as judged by a ruler), so they are effectively two-dimensional, and because the strain composition of our colonies does not vary with height inside the colony. We confirmed that strain composition does not vary with height by using a confocal microscope to probe the internal structure and also by taking a pipette tip, scratching it through a sector, growing the cells touched by the tip in overnight culture, and verifying that plated single colonies from the culture were the same color as the sector.

### Measuring radial expansion velocities *u*_*i*_

**Fig 11:**
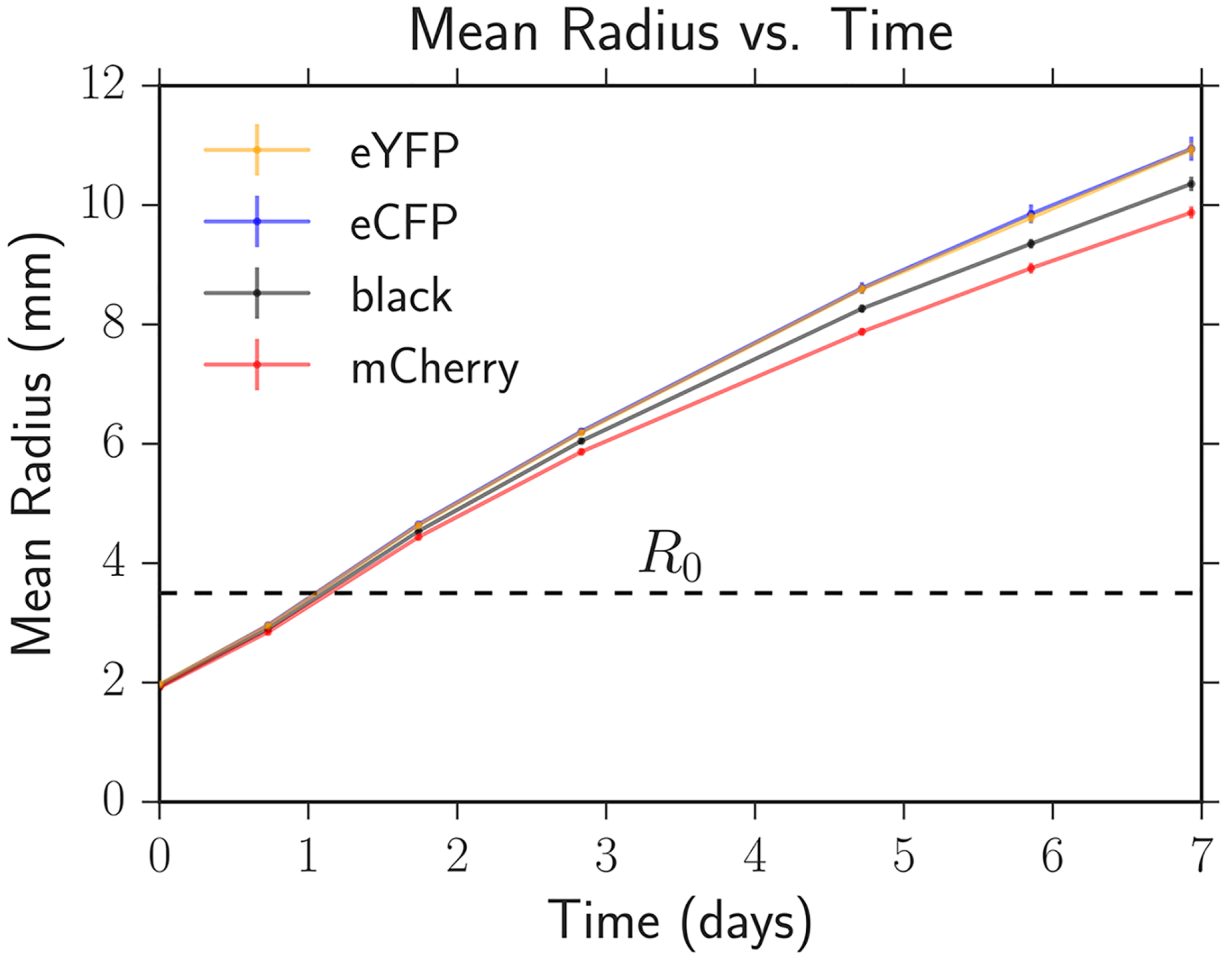
The average colony radius versus time for each strain on one of our three independent sets of agar plates. The error bars (comparable to symbol size at early times) are the standard errors of the mean calculated from 12 replicate expansions for each strain. The eYFP and eCFP strains had the fastest expansion velocities (data points overlap in the plot) followed by black and then mCherry. *R*_0_ is the radius at which expansions with competing strains typically demix into one color locally; *R*_0_ is approximately 1.75 times the initial inoculant radius of 2 mm (see Fig 1).

We used the average expansion velocity of each strain for radii *R* > *R*_0_ as a proxy for selective advantage, similar to previous work [17, 35]. In three independent sets of experiments using different batches of agar plates (the main source of variability in our experiments), we measured the diameter of 12 expansions of each strain approximately every 24 hours following the protocol for range expansions with two or more strains. To account for biological variance, sets of four of the 12 colonies were created from independent single colonies; no statistical difference was seen between biological replicates. The diameters were determined by manually fitting a circle to a brightfield image of the expansion three times and averaging the measured diameters. Fig 11 shows the average radius increasing with time for each strain from one of our experiments. In every experiment, the eCFP and eYFP strains had the fastest expansion velocities (the respective datapoints overlap in Fig 11), followed by the black strain, and then finally the mCherry strain. The expansion velocity slowly decreased as a function of time; we attribute this to nutrient depletion in the plates.

The radial expansion velocity of each strain was obtained by using linear regression to fit the radius versus time for radii greater than *R*_0_. We calculated the average radial expansion velocity between the three sets of plates and reported its error as the standard error of the mean; see Table 1. Additionally, we quantified the dimensionless selective advantage of each strain relative to the slowest growing mCherry strain following [17] via *s*_*iR*_ = *u*_*i*_/*u*_*R*_ − 1 where the *R* indicates the mCherry strain (red) in each experiment. The selective advantages were consistent, within error, when we calculated the velocities *u*_*i*_ and *u*_*R*_ over different time intervals. We averaged *s*_*iR*_ across our three experiments and reported its error as the standard error of the mean as seen in Table 1.

The eCFP and eYFP strains had an average selective advantage of 9%, similar to the experiments of Weber et al. [35] which found, despite the fact that they used different *E. coli* strains and plasmids, that the expression of mCherry decreased the expansion velocity of their strains by approximately 15% in certain “fast growth” environmental conditions. Our black strain had an approximately 6% enhancement over the mCherry strain. Differences in radial expansion velocities of this magnitude have been used to study yeast *S. cerevisiae* and *E. coli* range expansions in the past [9, 17]. To investigate the source of this fitness defect, we took the plasmids from our original strains, inserted them into a different set of clonal DH5*α* cells, and inoculated the new eCFP, eYFP, and mCherry strains in equal proportions in a range expansion. We saw that the average mCherry fraction decreased by 10% at a radius expanded of *R* = 10 mm, matching the results of Fig 2, suggesting that the presence of the plasmids was responsible for the fitness defect.

From Table 1, it is clear that the variance in *s*_*iR*_ was large between different sets of agar plates. Although *s*_*iR*_ varied significantly, the order of expansion velocities between the strains was consistent; the eCFP and eYFP strains always expanded faster than the black strain which expanded faster than the red strain. Importantly and in stark contrast to *s*_*iR*_, the demixing radius *R*_0_, wall velocities $v_{w}^{ij}$, and diffusion coefficient *D*_*w*_ were very consistent between sets of plates (measured below), resulting in consistent evolutionary dynamics between our competing strains.

### Comparing well-mixed fitness to fitness from expansion velocities

To test if the radial expansion velocity differences were related to the basal growth rates of our strains in liquid-culture, we competed all of our strains against mCherry in 10mL flasks of 2xYT growth media. We created three independent replicates of each pairwise competition (9 tubes in total) in the flasks by inoculating 60% of mCherry and 40% of the other strain in mid-log phase. We passaged saturated *E. coli* samples into new media every 24 hours; we determined the population composition of each flask using a BD LSR Fortessa FACS machine when the cells were passaged. We competed the strains for 72 hours, corresponding to approximately 45 generations (doubling times). We found that every strain grew faster than mCherry in every replicate in liquid culture as judged by a decrease in fraction of mCherry over time. Following previous work [43], we used the decrease in mCherry fraction to determine the dimensionless “well-mixed” selective advantage $s_{iR}^{wm}=g_{i}/g_{R}-1$ of the strain competing against it, where *g*_*i*_ is the growth rate of strain *i* and *g*_*R*_ is the growth rate of mCherry. We list the measured values of $s_{iR}^{wm}$ in Table 1.

The radial expansion velocity fitness *s*_*iR*_ did not agree with the liquid-culture fitness $s_{iR}^{wm}$ within error, in contrast to previous experiments with the yeast *Saccharomyces cerevisiae* [17]. However, every strain in liquid culture still grew faster than mCherry. Interestingly, in well-mixed culture, the black strain had the largest growth rate followed by eCFP and eYFP (they had the same growth rate) and then mCherry, disagreeing with the order of radial expansion velocities (where black expanded slower than eCFP and eYFP). As the growth rate differences were small, it is possible that additional factors allowed the eCFP and eYFP strains to expand faster than black on agar. Our *E. coli* switched from log to stationary phase in the 24 hour cycle; the changing environment may have resulted in a different order of fitnesses compared to the agar plates as well. Future work should investigate how the eCFP and eYFP strains expanded faster than the black strain despite a smaller basal growth rate and should also investigate how such small growth rate differences in liquid culture resulted in such large differences in radial expansion velocities on solid agar.

### Measuring the local fixation radius *R*_0_

When calibrating our model to experiment, the precise value of *R*_0_ did not matter as long as each strain’s local fraction could be accurately measured at that radius. Therefore, to maximize the length over which we could quantify range expansion growth, we defined the local fixation radius *R*_0_ as the minimum radius where our image analysis package became accurate. For *R* < *R*_0_, our package predicted equal fractions of each strain due to the overlap of each channel in the homeland (see Fig 10). Therefore, to determine *R*_0_, we inoculated radial expansions with three strains in unequal proportions; we used 10% of two strains and 80% of another. The minimum radius where the fractions agreed with their inoculated values was *R*_0_ = 3.50 ± 0.05 mm as seen in Supplementary S6 Fig. We found that this value of *R*_0_ worked for all colonies.

### Measuring the domain wall diffusion coefficient *D*_*w*_

Past work has found that *E. coli* colony domain walls fluctuate diffusively in certain conditions [18] and super-diffusively in others [8]. In our expansions, the domain walls appeared to fluctuate super-diffusively (as judged by tracking the position of domain walls and determining their variance vs. length expanded), but we were able to successfully fit the evolutionary dynamics using a diffusive theory. Creating a super-diffusive theory to describe the evolutionary dynamics of our system is beyond the scope of this paper. To obtain an effective diffusion constant *D*_*w*_ and to test if the diffusive approximation adequately described our experimental dynamics, we fit the neutral Voter model’s prediction of heterozygosity. The heterozygosity is the probability that two points separated by an angle of *ϕ* at a length expanded of *L* = *R* − *R*_0_ are occupied by *different* strains and is thus a measure of spatial genetic diversity. The neutral Voter model’s prediction of heterozygosity can be given in terms of the two-point correlations used in the main text or can be explicitly written as (see S1 Appendix)

$$\begin{array}{c} H\left(\phi ,L\right)=\sum_{i}\sum_{j\neq i}F_{ij}\left(\phi ,L\right)=H_{0}\mathrm{erf}[\sqrt{\left(1+R_{0}/L\right)}|\phi /\phi_{c}|]. \end{array}$$ (11)

*H*_0_ = *H*(*ϕ*, *L* = 0) is the heterozygosity when *L* = 0 and $\phi_{c}=\sqrt{8D_{w}/R_{0}}$ is a characteristic angular correlation length (one of the key combinations of model parameters from the main text). For *q* colors inoculated in equal fractions, *H*_0_ = 1 − 1/*q*.

**Fig 12:**
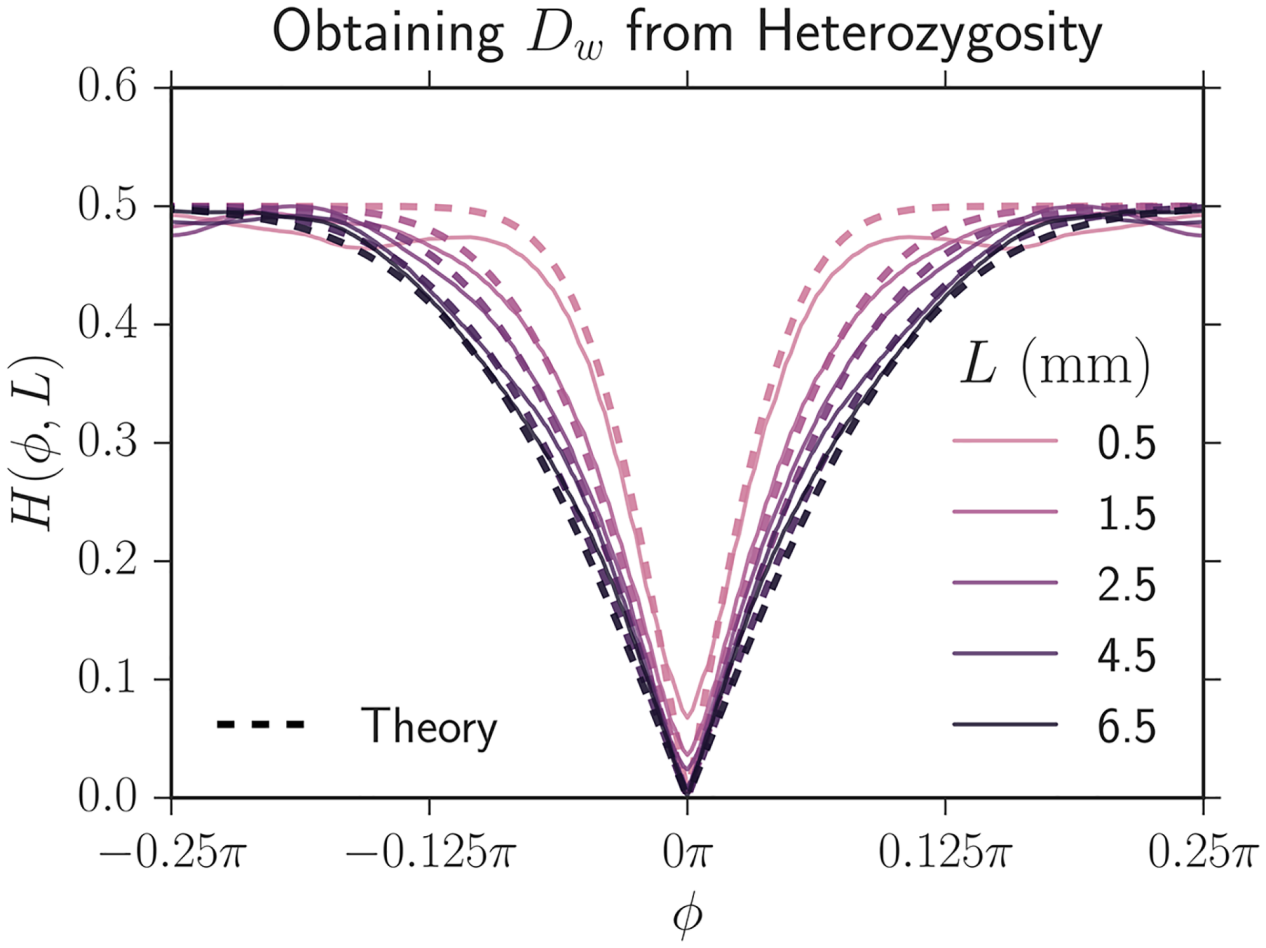
The heterozygosity correlation function *H*(*ϕ*, *L*) (solid lines) obtained by averaging the results of 14 neutral eCFP and eYFP expansions from one set of agar plates at a variety of expansion distances *L* = *R* − *R*_0_. The dashed lines are the theoretical fits of the heterozygosity with a constant *D*_*w*_ = 0.100 ± 0.005 mm. The theoretical curves track our experimental data, suggesting that a diffusive approximation to domain boundary motion is justified.

We fit *H*(*ϕ*, *L*) to our experimentally measured heterozygosity of two neutral strains (eCFP and eYFP) on three independent sets of agar plates each with 14 range expansions. We averaged the heterozygosity at each *L* as can be seen in Fig 12 (error bars were omitted for readability; the same figure with error bars can be found as Supplementary S7 Fig). As we had previously measured *R*_0_ = 3.50 ± 0.05 mm, and *H*_0_ = 1/2 for two neutral strains inoculated at equal fractions, *D*_*w*_ is the single free parameter in eq (11). We consequently fit *D*_*w*_ at each *L* with non-linear least-squares, averaged the *D*_*w*_ from the three independent experiments, and found *D*_*w*_ = 0.100 ± 0.005 mm; the reported error is the standard error of the mean between the experiments. The value of the diffusion constant is on the same order of magnitude as that from previous work [18].

Fig 12 shows the Voter model’s fit (dashed lines) together with the experimental heterozygosity (solid lines) for one set of plates using our values of *D*_*w*_ and *R*_0_. The fit closely matches the experimental heterozygosity suggesting that a diffusive description of *E. coli* domain motion is justified. We use this value of *D*_*w*_ for all strains. In principle, *D*_*w*_ may depend on *ij*, the particular domain wall type. However, we checked that the measured value of *D*_*w*_ did not vary for our all *ij* (all strain) combinations by examining the variance in domain wall position versus length expanded; the variances agreed within error and were thus consistent with a constant *D*_*w*_. The two-point correlation functions in the main-text were well fit by a constant *D*_*w*_ as well. Unlike the Voter model and our simulations, the experimental heterozygosity at zero separation *H*(*L*, *ϕ* = 0) fails to vanish due to overlap between strains at domain boundaries; this effect is less pronounced at large radii because the effective angular width of boundaries decreased. The discrepancy between the theoretical and experimental heterozygosity is larger at small lengths expanded because the overlap between strains is larger; our image analysis is consequently less accurate.

### Measuring the domain wall velocities $v_{w}^{ij}$

**Fig 13:**
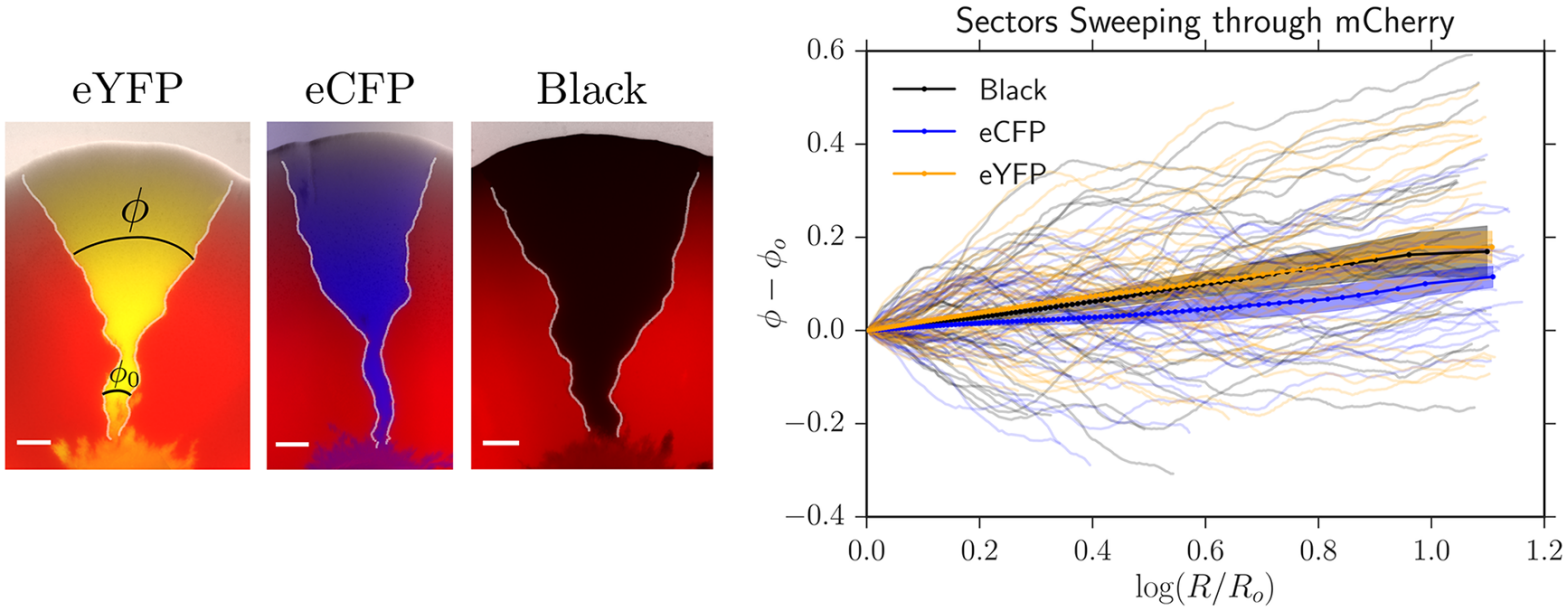
*Left:* Single sectors of our eYFP, eCFP, and black strains sweeping through the less fit strain mCherry. The scale bar is 1 mm. The white lines are the positions of the domain walls located with our image analysis package [34]. We tracked the angular growth of sectors sweeping through a less fit strain, *ϕ* − *ϕ*_0_, as a function of ln(*R*/*R*_0_) to obtain $v_{w}^{ij}$. *Right:* 40 traces of each strain sweeping through mCherry from one set of agar plates. The translucent lines are the individual traces, the solid lines are the mean angular growth 〈*ϕ* − *ϕ*_0_〉, and the shaded area is the standard error of the mean. The slope of the mean angular growth is $2v_{w}^{ij}$.

We used image analysis to directly quantify $v_{w}^{ij}$ from the angular growth of more-fit sectors. Characteristic single sectors of each strain sweeping through the mCherry strain can be seen on the left side of Fig 13. In radial expansions, more fit strains should, on average, sweep logarithmic spirals through less fit strains at large lengths expanded, as verified in yeast expansions [17]. It can be shown that the average angular width of a sector of strain *i* sweeping through strain *j* is given by (see S1 Appendix for more details)

$$\begin{array}{c} \left\langle \phi -\phi_{0}\rangle =2v_{w}^{ij}\ln(R/R_{0}\right) \end{array}$$ (12)

where *ϕ* is the angular width at radius *R* and *ϕ*_0_ is the initial angular width of the domain at *R*_0_. $2v_{w}^{ij}$ can thus be extracted from the slope of a linear regression fit of 〈*ϕ* − *ϕ*_0_〉 vs. ln(*R*/*R*_0_) as seen on the right side of Fig 13.

By tracking domain walls directly, we found that more fit strains (eCFP, eYFP, black) swept through the less fit mCherry strain with a wall velocity of $v_{w}^{iR}=0.06\pm 0.02$. We could not accurately measure the wall velocity of the eCFP and eYFP strains sweeping through the black strain. The wall velocity was significantly smaller than expected from the basal independent expansion velocities of our strains (Table 1); potential explanations for this phenomenon are discussed in S2 Appendix. The magnitude of the velocities were consistent between experiments (using 40 single sectors on three sets of plates) but were too imprecise to be predictive in our models.

### Simulation methods

**Fig 14:**
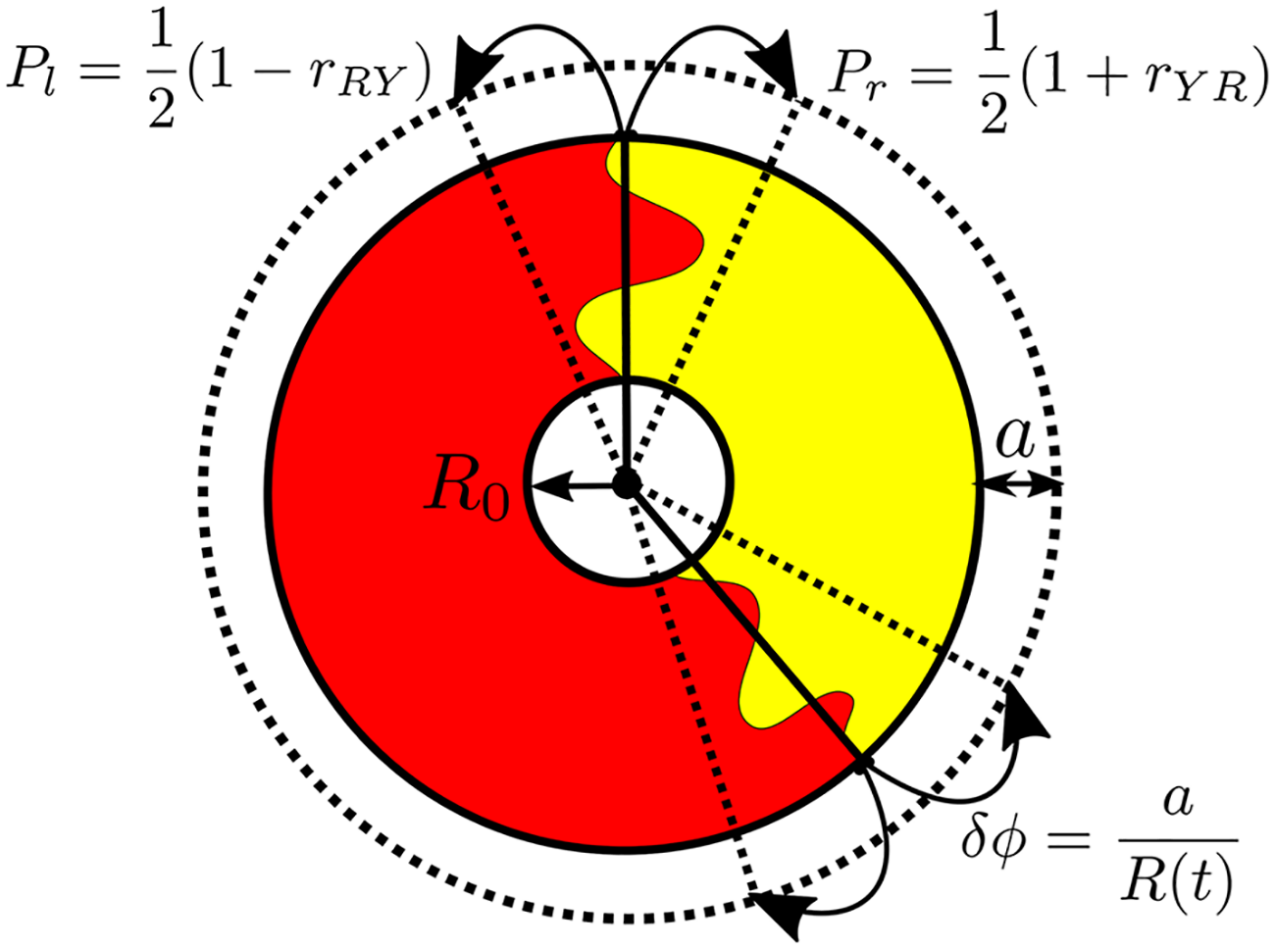
A schematic of the simulation procedure for a radial expansion. The initial population is a circle of cells of radius *R*_0_ = *N*_0_*a*/2*π*, where *N*_0_ is the initial number of cells and *a* is a cell width. During each time step (generation), the expansion advances a distance *a*; the radius consequently grows according to *R*(*t*) = *R*_0_ + *at* where *t* is the time in generations. The dashed circle shows the population after one generation time. Each domain wall position is tracked on the inflating ring (solid lines). At each time step, domain walls (two shown) hop to the left or right with probability *P*_*l*_ and *P*_*r*_, respectively, with an angular jump length *δϕ* ≡ *a*/*R*(*t*), and the position is updated (dashed lines). After each domain wall movement, the time in generations is incremented by 1/*N* where *N* is the number of domain walls present. For a *linear* simulation, the radius is simply not inflated in time, i.e. *R*(*t*) = *R*_0_.

Lattice simulations of range expansions, especially radial ones, can suffer from artifacts arising from the preferred directions of the lattice. It is possible to use an amorphous Bennett model lattice [44] to mitigate some of these effects [32]. Instead, we developed a simple off-lattice method that treats the domain walls as annihilating and coalescing random walkers moving along the edge of an inflating ring. The basic idea of the simulation is illustrated in Fig 14. We incorporate both the random, diffusive motion of the domain walls as well as deterministic movement due to selection. The radial expansion procedure is most easily understood by first considering a linear range expansion simulation for which the simulation steps are as follows:

1. Create a line of *N*_0_ microbes of width *a* at the linear frontier. Assign each microbe one of the *q* potential alleles.
2. Identify genetic domain walls by locating neighbors with different alleles; assign type *ij* to each wall where *i* and *j* are the strains to the left and right respectively. Assign a relative “growth rate” *r*_*ij*_ to each wall characterizing the bias in the probability that strain *i* divides into new territory before strain *j*. Two such domain walls are shown in a radial expansion in Fig 14.
3. Choose a wall at random and move it a distance *a* (the width of the cells) to the left or right; this represents the competition to reproduce and occupy new space at the population frontier. We use periodic boundary conditions for the domain wall positions along the line, meaning that the domain walls live on a circle, as shown for the radial case in Fig 14.
  (a) Jump to the right with a probability of $P_{r}=\frac{1}{2}(1+r_{ij})$ or to the left with probability $P_{l}=\frac{1}{2}(1-r_{ij})$. Note that domain walls separating neutral strains (*r*_*ij*_ = 0) will jump to the left or right with equal probability and that *r*_*ij*_ ≤ 1.
  (b) If the hopping domain wall collides with another wall, react the walls instantaneously with an appropriate annihilation or coalescence depending on whether the leftmost and rightmost strains are the same or different respectively.
4. Increment the elapsed time by Δ*t* = 1/*N* generations, where *N* is the number of domain walls at the beginning of the jump, and increment the length expanded by the colony by Δ*L* = *a*Δ*t* = *a*/*N*, where *a* (the cell width) is the distance that the colony expands per generation. Note that this length increment Δ*L* could also be some set by a different length scale *d*, i.e. Δ*L* = *d*/*N* (in our experiments, colonies typically expand further than a cell width during each generation due to growth behind the front). This does not change our analysis and we choose *d* = *a* for simplicity.
5. Repeat steps 3 and 4 until no domain walls remain or until the simulation has run for the desired number of generations.

Our simulation’s diffusion coefficient per length expanded, characterizing the random motion of the domain walls, can be shown to be *D*_*w*_ = *a*/2 when *r*_*ij*_ is small while its wall velocity per length expanded, characterizing the deterministic displacement of domain walls due to selection, can be shown to be $v_{w}^{ij}=r_{ij}\leq 1$.

Our algorithm, thus far simulating only linear expansions, can easily be extended to simulate radial geometries. To incorporate the radially inflating perimeter, we note that a domain wall at a radius *R* will jump an angular distance of *δϕ* = *a*/*R*, as shown in Fig 14. As the radius of our experimental expansions increases approximately linearly with generation time, we describe its radius as *R* = *R*_0_ + *at*. We thus account for inflation by using a time-varying angular jump length of

$$\begin{array}{c} \delta \phi \left(t\right)=\frac{a}{R_{0}+at}. \end{array}$$ (13)

If there are *N*_0_ individuals at the frontier, *R*_0_ is given by *R*_0_ = *N*_0_*a*/(2*π*). This modification of the domain wall step size *δϕ* is the only difference between the radial and linear cases!

**Fig 15:**
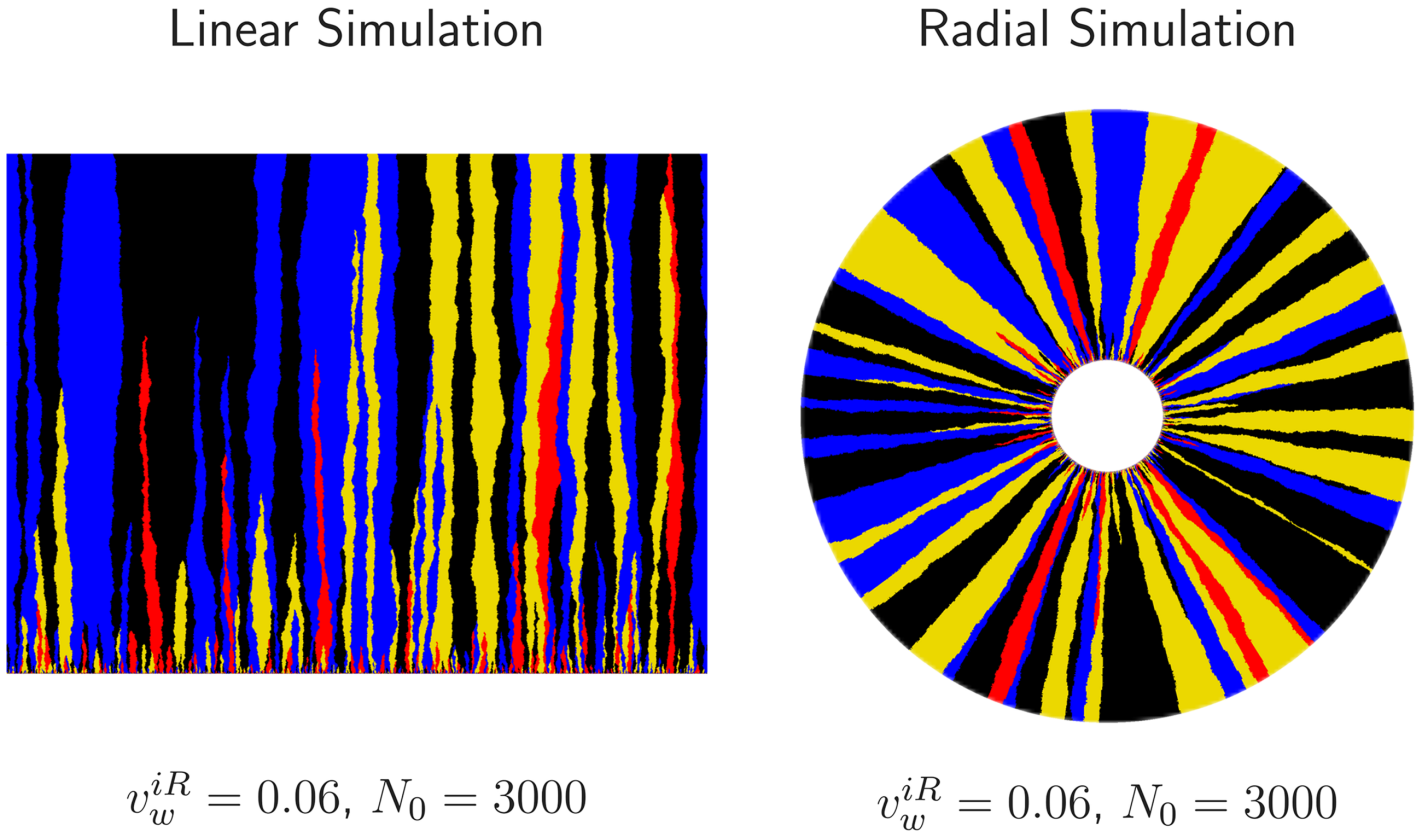
Simulations of linear (left) and inflating (right) range expansions grown for approximately 2000 generations. The black, yellow, and blue strains all sweep through the red strain with a wall velocity of $v_{w}^{iR}=0.06$. The initial concentration of all four strains was equal and *N*_0_ = 3000 was the number of cells at the initial front of both expansions. Note that the black, yellow, and blue sectors dominate over the red sectors at the end of these expansions.

In contrast to algorithms that follow the position and state of every organism at the front of a colony, our algorithm only tracks the positions of domain walls and is consequently much faster per generation as the sectors coarsen, allowing for simulations of larger colonies. Fig 15 displays a radial and linear simulation with three neutral colors and a fourth red color with a selective disadvantage comparable to our experiments. We check that our simulation correctly reproduces the behavior of a single more fit domain wall sweeping through a less fit strain as we vary simulation parameters in Supplementary S8 Fig. Our implementation of this algorithm and examples of how to use it are available on GitHub [34].

## Supporting information

S1 Appendix Supplemental theory. (PDF)

S2 Appendix Quantifying the discrepancy between radial expansion velocity and wall velocity. (PDF)

S1 Fig Average cumulative annihilations and coalescences for two, three, and four strains. All strains were inoculated in equal fractions except for the experiment with 10% of eCFP, 10% of eYFP and 80% of mCherry. The annihilation and coalescence rates (the slope of the respective curves) decrease as radius increases as there are less domain walls due to previous collisions and also because inflation decreases the probability of two walls colliding per length expanded. As the number of colors increases, coalescences occur more often than annihilations.
(TIF)

S2 FigCollapse of *F*_*ij*_.We ran four simulations where we varied *v*_*w*_ (the velocity that the two other more fit strains swept through the less fit strain) and *R*_0_ such that $L_{s}=D_{w}/v_{w}^{2}$ changed but $\kappa =\sqrt{R_{0}/L_{s}}$ was fixed. Each simulation has a different symbol in the plot. We found that *F*_*ij*_ could be collapsed at the same *L*/*L*_*s*_ as long as *κ* remained fixed (we arbitrarily set it to *κ* = 0.4) and as long as the angular variable *ϕ* was rescaled by $\phi_{c}=\sqrt{8D_{w}/R_{0}}$. If *ϕ*_*c*_ approached the system size *ϕ*_*c*_ ≈ 2*π*, *F*_*ij*_ could not be collapsed onto the above curves due to finite size effects. Note that even though we only show *F*_*RR*_, all correlation functions *F*_*ij*_ could be collapsed using this procedure.(TIF)

S3 Fig Collapsed average fraction and annihilation asymmetry on a linear scale. Identical to Fig 6 except the *y*-axis of *F*(*L*/*L*_*s*_, *κ*) is placed on a linear scale, which may be useful for comparison with experiments.
(TIF)

S4 FigCollapse of average fraction and annihilation asymmetry.We simulated three competing strains and arbitrarily chose a fixed set of $\kappa^{ij}=\sqrt{R_{0}/L_{s}^{ij}}=\sqrt{R_{0}(v_{w}^{ij}{)}^{2}/D_{w}}$ to reflect differing selective advantages between the strains. We set the initial radius of the expansion *R*_0_ = *N*_0_*a*/(2*π*) to *N*_0_ = {200, 2000, 20000} in three simulations and altered $v_{w}^{ij}$ to tune the set of *κ*^*ij*^’s to their fixed values. We found that the dynamics collapsed as long as *L* was rescaled by any selection length scale in the system, i.e. $L/L_{s}^{ij}$ (we chose to use $L_{s}^{YR}$). The diamond simulation, corresponding to *N*_0_ = 200, deviated slightly from the other simulations because of finite size effects (i.e. when *ϕ*_*c*_ ∼ 2*π*).(TIF)

S5 Fig Image processing artifacts introduced by using a non-fluorescent (i.e. black) strain. To estimate the image analysis artifacts introduced by using a non-fluorescent, black strain we performed an experiment with three fluorescent strains (eCFP, eYFP, and mCherry in equal initial proportions) and analyzed the data twice: once where we included all three fluorescent channels and once where we excluded the eCFP channel and treated it as if it were a black strain. We compared the black-substituted average fractions *F*_*i*_ (the dashed lines) to the real fractions as a function of radius (the solid lines). At a small radius relative to *R*_0_ = 3.5 mm, the error from introducing a black strain was large; this is likely because we defined black as the absence of any other channels and channels typically had large overlaps close to the homeland. At large radius, the error from introducing a black strain was negligible.
(TIF)

S6 Fig Determining *R*_0_. To fit the radius *R*_0_ where our image analysis package became accurate, we inoculated 80% of mCherry, 10% of eCFP, and 10% of eYFP in 10 range expansions and tabulated the average fraction of each strain. The inoculated fractions are illustrated by dashed lines. As seen in the plot, at a radius of approximately *R*_0_ = 3.50 ± 0.05 mm the measured average fractions were closest to the inoculated fractions. Our image analysis package inaccurately predicted fractions in the homeland because of significant overlap between the strains.
(TIF)

S7 Fig Error bars when fitting *D*_*w*_. The same as the right side of Fig 12 except with error bars; the shaded areas are the standard error of the mean.
(TIF)

S8 FigConfirming simulation accuracy.We simulated a single fit sector sweeping through a less fit strain. It is expected that the fit strain sector dynamics satisfy 〈*ϕ* − *ϕ*_0_〉 = 2*v*_*w*_ ln(*R*/*R*_0_) and Var(*ϕ*) = 4*D*_*w*_(1/*R*_0_ − 1/*R*), as seen in S1 Appendix. To test that our simulation appropriately reproduced this behavior, we quantified the average angular growth 〈*ϕ* − *ϕ*_0_〉 and angular variance Var(*ϕ*) as we varied the simulation parameters *N*_0_ (initial number of cells), *r* (selective advantage of the fitter strain), and *d* (distance the colony expanded each generation). The cell width, *a*, was kept constant. These parameters relate to the sector dynamics via *D*_*w*_ = *a*^2^/(2*d*), $v_{w}^{ij}=ar_{ij}/d$, and *R*_0_ = (*N*_0_*a*)/(2*π*). We confirmed that both the average angular growth 〈*ϕ* − *ϕ*_0_〉 and angular variance Var(*ϕ*) had the correct functional form and dependence on the microscopic parameters (the dashed black line). In the main text, we used *d* = *a* for simplicity.(TIF)
