# Supplementary material for: Genetic drift and selection in many-allele range expansions
Source: PLoS Comput Biol. 2017 Dec 1;13(12):e1005866. doi: 10.1371/journal.pcbi.1005866 (PMC5728587; doi:10.1371/journal.pcbi.1005866)
Supplement: S1 Appendix — (PDF) [file pcbi.1005866.s001.pdf]

Appendix S1: Supplemental theory

As discussed in the introduction, it is difficult to make analytical progress when modeling a many-allele range expansion as a line of annihilating and coalescing domain walls subject to diffusion and deterministic, selection-induced motion. This is because the moment hierarchy of an equivalent system, the q-color Voter Model, does not close [1]. Much is understood, however, about the neutral dynamics of many annihilating and coalescing walls; analytical predictions exist for spatial correlation functions [2] and relative annihilation and coalescence rates [2–4]. In addition, results for the dynamics of a monoclonal single sector (bordered by domain walls) of a more fit strain sweeping through a less fit strain are available [1, 5, 6]. In this section, we review previous theoretical work and introduce theoretical advances relevant to the main text.

The Stepping-Stone and Voter models with selection

The population dynamics of range expansions with flat fronts can be modeled as the evolution of a one-dimensional line of individuals subject to growth/replication, death, and diffusion with the constraint of a constant population density [1]. Two well-studied microscopic models of the dynamics are the Voter model [6] and the Stepping Stone model [7]. The Voter model is equivalent to a one-dimensional q-state Potts model [8, 9] governed by zero-temperature Glauber dynamics [10]; individuals in the population are replaced by one of their neighbors with a constant probability per time. The stepping stone model (prior to taking a continuum limit) assumes that there are many connected "islands" that individuals diffuse to and from governed by Moran reproduction [11]; each of these islands has a population size of N. The Voter model can be viewed as a stepping stone model where the local population of each island is $N = 1$.

When either model is coarse-grained in space, the resulting stochastic differential equation governing the evolution of $f_i(\vec{x}, t)$, the fraction of strain i at position $\vec{x}$ at time t, is the same, but with different values for the model parameters and boundary conditions. The stochastic differential equation for an arbitrary number of competing strains along the line is

$$\frac{\partial f_i}{\partial t} = D_w \nabla^2 f_i + [s_i - \langle s \rangle] f_i + \sum_j [\delta_{ij} - f_i] \sqrt{D_g f_j} \zeta_j(\vec{x}, t), \tag{S1.1}$$

which is a spatial generalization of the equation studied by Good et al. [12]. Here, δ_{ij} is the Kronecker delta function ($\delta_{ij} = 1$ if $i = j$ and $\delta_{ij} = 0$ otherwise), D_w is the spatial diffusion coefficient of each strain and is the same D_w as the one used in the main text, s_i is the dimensionless fitness of strain i, $\langle s \rangle = \sum_j s_j f_j$ is the mean fitness of all strains locally, and D_g parameterizes genetic drift. The function $\zeta_i(\vec{x}, t)$ is a white noise random variable with the properties $\langle \zeta_i(\vec{x}, t) \rangle = 0$ and $\left\langle \zeta_i(\vec{x}, t) \zeta_j(\vec{x'}, t') \right\rangle = \delta_{ij} \delta(\vec{x} - \vec{x'}) \delta(t - t')$, where these noises are interpreted in the Itô sense [13]. The noise term on the far-right captures fluctuations due to the limited population size. Equation (S1.1) reduces to standard equations in the literature [1] when describing with two competing strains. Unfortunately, equation (S1.1) becomes analytically intractable when multiple strains and selective advantages are present because of the closure problem: $\frac{\partial}{\partial t} \langle f_i \rangle$ depends on $\langle f_i f_j \rangle$, and $\frac{\partial}{\partial t} \langle f_i f_j \rangle$ depends on $\langle f_i f_j f_k \rangle$, etc. The moment hierarchy does not close.

Neutral correlation functions

Much is known about equation (S1.1) in the neutral case where all $s_i = 0$ as discussed in the main text [1]. Unsurprisingly, it can be shown that the average fraction $F_i = \langle f_i \rangle$ (the bracket indicates an average over many expansions) equals the initial inoculated fraction F_i^0 at all times for an initially well-mixed inoculant. Although the average fraction is constant in the neutral case, the two-point correlation function broadens due to the coarsening of genetic domains. Upon adopting polar coordinates for radial expansions and letting $L = ut = R - R_0$ where u is the radial expansion velocity, it can be shown, using equation (S1.1) when all $s = 0$, that the dynamics of the average two-point correlation functions $F_{ij}(\phi, L) = \langle f_i f_j \rangle$ are governed by (in polar coordinates),

$$\frac{\partial}{\partial L} F_{ij}(\phi, L) = \begin{cases} i = j, & \dfrac{2D_w}{(R_0+L)^2} \dfrac{\partial^2}{\partial \phi^2} F_{ii} + \dfrac{D_g}{R_0+L} \delta(\phi) \left[F_i - F_{ii} \right] \\ i \neq j, & \dfrac{2D_w}{(R_0+L)^2} \dfrac{\partial^2}{\partial \phi^2} F_{ij} - \dfrac{D_g}{R_0+L} \delta(\phi) F_{ij}. \end{cases} \tag{S1.2}$$

where ϕ is the angular distance between points at the frontier and $\delta(\phi)$ is a Dirac delta function. For the Voter model with deme size $N = 1$, the boundary conditions are given by $F_{ij}(L=0) = F_i^0 F_j^0$, $F_{i=j}(\phi = 0) = F_i$, and $F_{i\neq j}(\phi = 0) = 0$; these conditions make the delta functions $\delta(\phi)$ disappear. F_i^0 and F_j^0 are the initial inoculated fractions of strains i and j. Solving these equations by making a "conformal time" substitution [6, 14, 15] yields

$$F_{ij}(\phi, L) = \begin{cases} i \neq j, & F_i^0 F_j^0 \operatorname{erf}\left[\sqrt{1 + R_0/L}\, |\phi/\phi_c| \right] \\ i = j, & F_i^0 \left\{ 1 - \left(1 - F_i^0\right) \operatorname{erf}\left[\sqrt{1 + R_0/L}\, |\phi/\phi_c| \right] \right\} \end{cases} \tag{S1.3}$$

where the characteristic angular width of F_{ij} is given by $\phi_c = \sqrt{8D_w/R_0}$; again note that D_w is the same as that from the main text. Figure A contains plots of $F_{ij}(\phi, L)$ for both $i \neq j$ and $i = j$. As $L \to \infty$, F_{ij} approaches a constant shape given by the error function because inflation will eventually completely dominate the diffusive motion of boundary walls which brings coarsening of genetic domains to a halt. When $\phi \gg \phi_c$, we have $F_{ij} \approx F_i^0 F_j^0$, because the different genetic regions become uncorrelated. Note that if ϕ_c approaches 2π, this limit is impossible to achieve and the correlation function will not factorize. These neutral results for $F_{ij}(\phi, L)$ tabulated above can be used as a null model when we introduce selection.

From the $F_{ij}(\phi, L)$ above, we define the heterozygosity correlation function as [1]

$$H(\phi, L) = \sum_i \sum_{j \neq i} F_{ij}(\phi, L) = H_0 \operatorname{erf}\left[\sqrt{(1 + R_0/L)}\, |\phi/\phi_c| \right] \tag{S1.4}$$

where H_0 is the heterozygosity at $L = 0$, or $H_0 = \sum_i F_i^0 (1 - F_i^0)$ (this assumes that that the initial condition is a well-mixed inoculation with some initial fraction F_i^0) . The heterozygosity can be thought of as the probability that two points separated by an angle of ϕ at a length expanded of L are occupied by different strains; it is a spatial measure of genetic diversity. This result is used to determine the wall diffusion constant D_w in the Measuring D_w section in the Materials and Methods section.

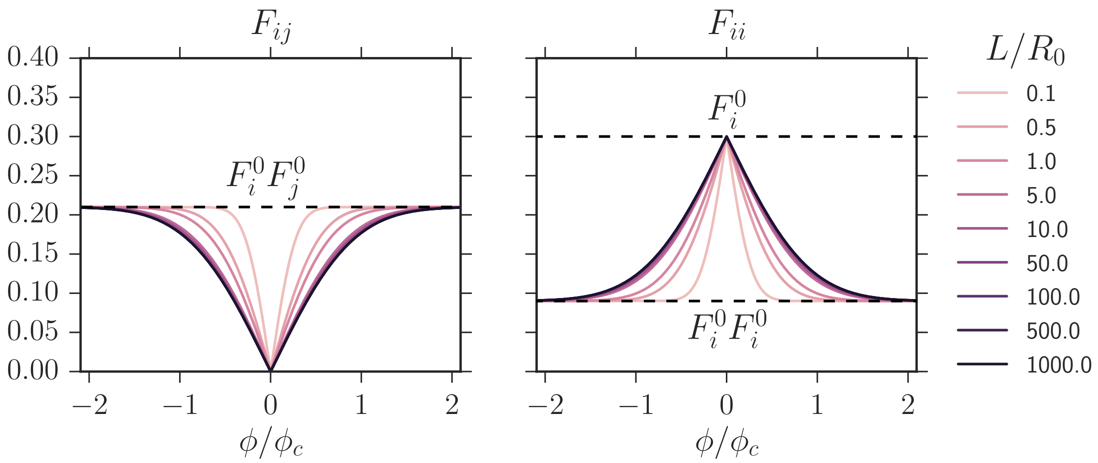

Fig A. Voter model predictions for $F_{ij}(\phi, L)$ from eq. (S1.3) for $i \neq j$ on the left and $i = j$ on the right. F_i^0 and F_j^0, the initial inoculated fractions of strains i and j, were set to 0.3 and 0.7 respectively. The product $F_i^0 F_j^0$ determines the asymptote of the correlation function for large angular separation. Note that $L/R_0 \geq 0$.

Neutral annihilation and coalescence probabilities

Upon collision, the diffusing domain walls either annihilate or coalesce as illustrated in Figure 1 of the main text. Upon regarding these genetic boundaries as world lines of chemical species, these processes can be described using the language of chemical reactions,

$$\textbf{Annihilation: } W_{ij} + W_{ji} \to \emptyset$$
$$\textbf{Coalescence: } W_{ij} + W_{jk} \to W_{ik} \quad (i \neq k),$$

where W_{ij} is a domain wall such that the strain on the left is of type i and the strain on the right is of type j. Note that the inner indices of colliding domain walls are always identical, because two neighboring domain walls always have a common strain between them.

Although little is known about the relative rates of annihilations and coalescences in the presence of selection, analytical results are available for the neutral case ($v_w^{ij} = 0$). If q (an integer) neutral alleles are inoculated at random locations with *equal* initial proportions on a one-dimensional lattice, the probability of obtaining an annihilation per domain wall collision is given by

$$P_A = \frac{1}{q-1} \tag{S1.5}$$

and the probability of obtaining a coalescence per collision is given by [2–4]

$$P_C = \frac{q-2}{q-1}. \tag{S1.6}$$

One can easily derive these formulas; given that strain i is to the left of two colliding domain walls and strain j is between them, we can ask: "what is the probability that strain k to the right of the walls is the same as strain i (annihilation) or is *not* strain i or j (coalescence)?" Note that although the rate of annihilations and coalescences decreases with time due to coarsening, the probabilities per collision $P_{A,C}$ are independent of the length expanded L. In the presence of

selection, however, the average global fraction of each strain will change with length expanded, making P_A and P_C L-dependent.

To succinctly quantify the difference between the annihilation and coalescence probabilities, we define the "annihilation asymmetry" $\Delta P(L) = P_A(L) - P_C(L)$ as the difference in probability of obtaining an annihilation versus a coalescence per collision at a distance expanded of L. If q neutral colors are inoculated in equal fractions, we find, using equations (S1.5) and (S1.6),

$$\Delta P = \frac{3-q}{q-1}. \tag{S1.7}$$

To determine how unequal global fractions of each neutral strain alters P_A and P_C and thus ΔP, we write P_A and P_C in terms of P_{ijk}, the probability that a collision between domain walls W_{ij} and W_{jk} occurred per collision:

$$P_A = \sum_{i}^{q} \sum_{j \neq i}^{q} P_{iji}, \tag{S1.8}$$

$$P_C = \sum_{i}^{q} \sum_{j \neq i}^{q} \sum_{k \neq i,j}^{q} P_{ijk}. \tag{S1.9}$$

We expect that for q neutral colors, the chance of a particular color combination in a collision ijk with color i on the left and color k on the right and j in the middle to be proportional to the product $F_i^0 F_j^0 F_k^0$ of the initial color fractions. We therefore expect that

$$P_{ijk} = \frac{Z_{ijk}}{Z_{\text{total}}} = \frac{F_i^0 F_j^0 F_k^0}{Z_{\text{total}}} \tag{S1.10}$$

where $Z_{ijk} = F_i^0 F_j^0 F_k^0$ and the normalization constant is $Z_{\text{Total}} = \sum_i^q \sum_{j \neq i}^q \sum_{k \neq j}^q Z_{ijk}$, i.e. the sum of all Z_{ijk}.

Using the simulations described in the Simulation methods section, we checked eq. (S1.10) for $q = 3$ neutral strains. The left side of Figure B displays the simulated values of P_{ijk} and our theoretical predictions for three neutral strains inoculated with initial fractions $\{F_1 = 0.1, F_2 = 0.3, F_3 = 0.6\}$ in a linear range expansion; our theoretical predictions, represented by black dashed lines, match the simulation results. As our predictions for P_{ijk} were correct, our predictions for P_A and P_C were also correct as they were composed of sums of P_{ijk}. Inflating simulations with the same F_i also returned the same values of P_{ijk} and thus P_A and P_C. Inflation changes the rate at which annihilations and coalescences occur, but not their relative proportions.

As discussed in the main text, to efficiently quantify the difference between the annihilation and coalescence probabilities, we defined the "annihilation asymmetry" $\Delta P(L) = P_A(L) - P_C(L)$ as the difference in probability of obtaining an annihilation vs. a coalescence in a given collision at a distance expanded of L. In the neutral case, ΔP is independent of L. The right side of Figure B displays a ternary diagram illustrating all possible values of ΔP that can be reached when inoculating $q = 3$ neutral colors in different proportions. The blue dot corresponds to the initial conditions where the P_{ijk} probabilities were calculated in the plot to its left. For a combination of three colors present in an expansion, ΔP is minimized when all three colors are inoculated in equal fractions and is maximized when one of the fractions of the three colors goes to zero. This is true for all q.

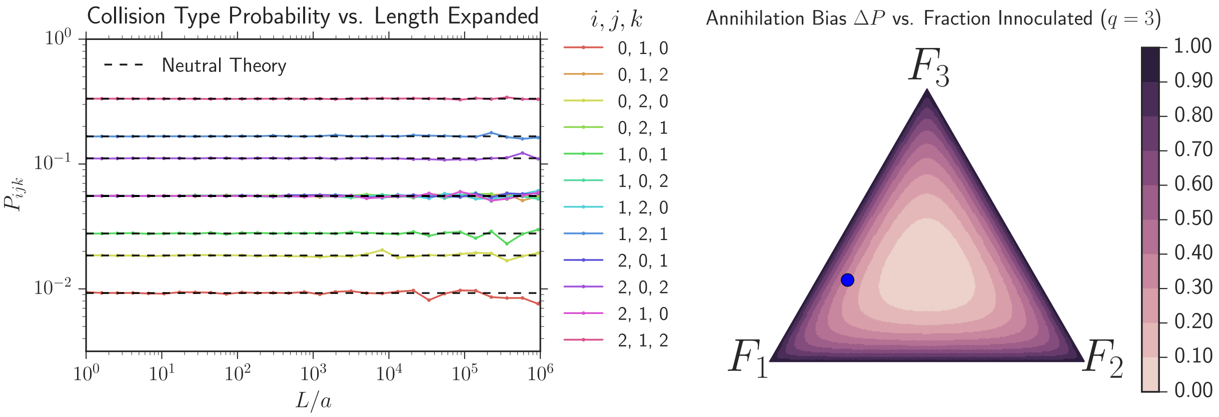

Fig B. *Left*: Probability P_{ijk} of a domain wall collision with color i to the left of the walls, color j between the walls, and color k to the right vs. length expanded. We simulated $q = 3$ neutral strains with initial fractions $\{F_1 = 0.1, F_2 = 0.3, F_3 = 0.6\}$ in a linear expansion, averaged the results of 1000 simulations, and calculated P_{ijk} (solid lines) and compared its value to that from eq. (S1.10) (dashed black lines). L/a is the length expanded divided by the cell size and is equivalent to the elapsed time in generations. The simulation confirms the predictions of eq. (S1.10). *Right:* The annihilation bias $\Delta P = P_A - P_C$, where P_A and P_C are the probabilities of obtaining an annihilation or coalescence event per domain wall collision respectively, calculated via eqs. (S1.8) and (S1.9) as a function of initial inoculated fractions for $q = 3$ neutral colors. ΔP is independent of length expanded for neutral strains. The large blue dot corresponds to the initial conditions that were used on the left. ΔP assumes its minimum value $\Delta P = 0$ when $q = 3$ colors are inoculated in equal fractions and is maximized on the boundaries of the ternary diagram corresponding to $\Delta P = 1$. Discrete colors were used to more clearly highlight the contours of ΔP.

Single sector dynamics with selection

We first review a simple phenomenological model [5, 6, 16] of the width w of a single sector of a more fit strain sweeping through a less fit strain incorporating both wall diffusion and deterministic motion due to selective differences. Let x be the position of one of the domain walls of a sector. We quantify a domain wall's displacement dx over a front expansion distance of dL by the parameters $2D_w = d\mathrm{Var}(x)/dL$ ($\mathrm{Var}(x) = \langle x^2 \rangle - \langle x \rangle^2$ is the variance), describing the diffusive motion of the wall, and $v_w = d\langle x \rangle/dL$, describing its deterministic motion, as discussed in the Introduction and illustrated in Figure 1 of the main text.

We first describe a linear range expansion and then extend our treatment to a radially inflating expansion. If we track the distance w between two walls that are sweeping through a less fit strain per length expanded L, as sketched on the right side of Figure 1 of the main text, the dynamics of w is given by

$$\frac{dw}{dL} = 2v_w + \sqrt{4D_w}\zeta(L) \quad \text{(S1.11)}$$

where $\zeta(L)$ is white noise with $\langle \zeta(L) \rangle = 0$ and $\langle \zeta(L)\zeta(L') \rangle = \delta(L - L')$ and should be interpreted in the Itô sense [13]. The factors of 2 in front of v_w and 4 in front of D_w arise because we monitor the distance between *two* domain walls. Note that we make the smooth front approximation that neglects the roughness of the expansion boundary. We assume that v_w is constant [17] and that

the domain wall motion is diffusive [6]. Within this approach, diffusive effects scale as $\sqrt{L}$ while deterministic effects scale as L; hence, at short expansion distances, diffusion dominates the sector width while at larger length scales, deterministic motion becomes apparent. A crossover length scale L_s follows by equating the deterministic average displacement (from the first term of (S1.11)) with the root mean squared displacement associated with the second term,

$$\underbrace{2v_w L_s}_{\text{Deterministic}} = \underbrace{\sqrt{4D_w L_s}}_{\text{Diffusive}} \quad \Rightarrow L_s = D_w/v_w^2. \tag{S1.12}$$

L_s is the distance that a linear expansion must expand in order for selection to become dominant over diffusive effects [1, 6, 18].

What changes in the radially expanding case? We now shift to radial coordinates. Upon setting $L = R - R_0$ where R_0 is the radius at which the alleles first fix, and denoting the angular width between the two sector boundaries as $\phi = w/R$, our phenomenological stochastic model becomes [6]

$$\frac{d\phi}{dL} = \frac{2v_w}{R_0 + L} + \sqrt{\frac{4D_w}{(R_0+L)^2}}\zeta(L). \tag{S1.13}$$

The mean and variance of the sector width ϕ are, with $R = L + R_0$,

$$\langle \phi - \phi_0 \rangle = 2v_w \ln\left(\frac{R}{R_0}\right) \qquad = 2v_w \ln\left(1 + \frac{L}{R_0}\right) \tag{S1.14}$$

$$\mathrm{Var}(\phi) = \left\langle \phi^2 \right\rangle - \langle \phi \rangle^2 = 4D_w\left(\frac{1}{R_0} - \frac{1}{R}\right) = \frac{4D_w}{R_0}\left[1 - \left(1 + \frac{L}{R_0}\right)^{-1}\right]. \tag{S1.15}$$

Eq. (S1.14) describes how the boundaries of the more fit domain sweep out a logarithmic spiral as the expansion inflates [6, 16, 17], and eq. (S1.15) shows that the effective angular diffusion coefficient decreases as the radius $R = R_0 + L$ increases. If one now equates the deterministic displacement of the boundaries to diffusive effects, in analogy with equation (S1.12), we find that the crossover between diffusive wandering of the sector width and a deterministic logarithmic sweep occurs at an expansion distance L_I that satisfies

$$\underbrace{\kappa \ln\left(1 + \frac{L_I}{R_0}\right)}_{\text{Deterministic}} = \underbrace{\sqrt{1 - \left(1 + \frac{L_I}{R_0}\right)^{-1}}}_{\text{Diffusive}} \tag{S1.16}$$

where the dimensionless parameter κ is an inflationary selective advantage, [6] $\kappa = \sqrt{R_0/L_s} = \sqrt{R_0 v_w^2/D_w}$ and L_I is the inflationary analog of L_s, the length scale at which selection dominates over diffusion on an inflating boundary [6, 16]. Fig. 5 of the main text displays the numerical solution of eq. (S1.16) for $L_I(\kappa)$.

The impact of κ on domain behavior is demonstrated visually in Figure C. Three simulations were conducted utilizing the algorithm from the Simulation methods Section with a more fit yellow strain sweeping through a less fit red strain. L_s was kept constant but κ was varied by altering the initial radius of each expansion. As κ decreases from right to left in Figure C, inflation plays a larger role and dramatically slowed down the sweep of the more fit strain.

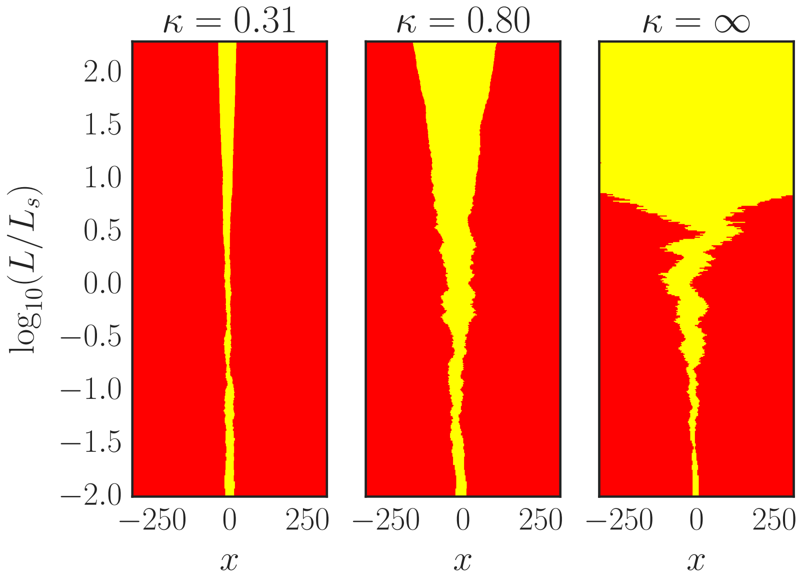

Fig C. The impact of κ on domain behavior. Three simulations were conducted using the algorithm from the Simulation methods section where a more fit yellow strain, initially occupying a width of 10 cells at the front (the horizontal axis is in units of cell widths), sweeps through a less fit red strain. As explained in the Simulation methods section, we accounted for inflation by appropriately decreasing the "jump size" of domain walls; this leads to the identification that $x = R_0\phi$ where ϕ is the angular position along the radially expanding front. As κ decreases from right to left, inflation plays a larger role and dramatically slows down sweeping through the less fit strain due to the decreasing domain wall jump length. $\kappa = \infty$ was obtained by not inflating the domain (a linear range expansion) with periodic boundary conditions; the expansion proceeds along a cylinder.

References

1. Korolev KS, Avlund M, Hallatschek O, Nelson DR. Genetic demixing and evolution in linear stepping stone models. Reviews of Modern Physics. 2010;82(2):1691–1718. doi:10.1103/RevModPhys.82.1691.

2. Masser TO, Ben-Avraham D. Correlation functions for diffusion-limited annihilation, $A + A \to A$. Physical Review E. 2001;64(6):062101. doi:10.1103/PhysRevE.64.062101.

3. Masser T, Ben-Avraham D. Kinetics of coalescence, annihilation, and the q-state Potts model in one dimension. Physics Letters A. 2000;275(5-6):382–385. doi:10.1016/S0375-9601(00)00622-8.

4. Derrida B, Zeitak R. Distribution of domain sizes in the zero temperature Glauber dynamics of the one-dimensional Potts model. Physical Review E. 1996;54(3):2513–2525. doi:10.1103/PhysRevE.54.2513.

5. Korolev KS, Xavier JB, Nelson DR, Foster KR. A Quantitative Test of Population Genetics Using Spatiogenetic Patterns in Bacterial Colonies. The American Naturalist. 2011;178(4):538–552. doi:10.1086/661897.

6. Lavrentovich MO, Korolev KS, Nelson DR. Radial Domany-Kinzel models with mutation and selection. Physical Review E. 2013;87(1). doi:10.1103/PhysRevE.87.012103.

7. Kimura M, Weiss GH. The Stepping Stone Model of Population Structure and the Decrease of Genetic Correlation with Distance. Genetics. 1964;49(4):561–576. doi:10.1093/oxfordjournals.molbev.a025590.

8. Potts RB, Domb C. Some generalized order-disorder transformations. Mathematical Proceedings of the Cambridge Philosophical Society. 1952;48(October):106. doi:10.1017/S0305004100027419.

9. Wu FY. The Potts model. Reviews of Modern Physics. 1982;54(I):235–268. doi:10.1103/RevModPhys.54.235.

10. Glauber RJ. Time-Dependent Statistics of the Ising Model. Journal of Mathematical Physics. 1963;4(2):294. doi:10.1063/1.1703954.

11. Moran PAP. Random processes in genetics. Mathematical Proceedings of the Cambridge Philosophical Society. 1958;54(01):60–71. doi:10.1017/S0305004100033193.

12. Good BH, Desai MM. Fluctuations in fitness distributions and the effects of weak linked selection on sequence evolution. Theoretical Population Biology. 2013;85:86–102. doi:10.1016/j.tpb.2013.01.005.

13. Gardiner C. Stochastic Methods: A Handbook for the Natural and Social Sciences. 4th ed. Springer; 2009. Available from: http://www.amazon.com/Stochastic-Methods-Handbook-Sciences-Synergetics/dp/3540707123.

14. Ali A, Grosskinsky S. Pattern formation through genetic drift at expanding population fronts. Advances in Complex Systems. 2009;13(03):349–366. doi:10.1142/S0219525910002578.

15. Ali A, Ball RC, Grosskinsky S, Somfai E. Scale-invariant growth processes in expanding space. Physical Review E. 2013;87(2):020102. doi:10.1103/PhysRevE.87.020102.

16. Hallatschek O, Nelson DR. Life at the front of an expanding population. Evolution; international journal of organic evolution. 2010;64(1):193–206. doi:10.1111/j.1558-5646.2009.00809.x.

17. Korolev KS, Müller MJI, Karahan N, Murray AW, Hallatschek O, Nelson DR. Selective sweeps in growing microbial colonies. Physical Biology. 2012;9(2):026008. doi:10.1088/1478-3975/9/2/026008.

18. Gralka M, Stiewe F, Farrell F, Möbius W, Waclaw B, Hallatschek O. Allele surfing promotes microbial adaptation from standing variation. Ecology Letters. 2016;19(8):889–898. doi:10.1111/ele.12625.
