# Supplementary material for: Genetic drift and selection in many-allele range expansions
Source: PLoS Comput Biol. 2017 Dec 1;13(12):e1005866. doi: 10.1371/journal.pcbi.1005866 (PMC5728587; doi:10.1371/journal.pcbi.1005866)
Supplement: S2 Appendix — (PDF) [file pcbi.1005866.s002.pdf]

Appendix S2: Quantifying the discrepancy between expansion velocity and wall velocity

When growing colonies of individual strains, we observed that the eCFP and and eYFP strains expanded faster than the black strain which in turn expanded faster than the mCherry strain (see Table 4). In addition, using the method to determine wall velocity from the two-point correlation function (fitting L_s^{ij}), we found that the eCFP and eYFP strains swept through the black strain which swept through mCherry when competing in the same expansion (see Table 3). These observations are consistent with a picture in which a larger expansion velocity difference leads to a larger wall velocity. Korolev et al. studied the connection between radial expansion velocity and wall velocity in detail for *S. cerevisiae.* Using geometric arguments, they argued that if the front of an expansion is sufficiently smooth, a domain wall bordering a strain i and a less fit strain j will have a constant wall velocity v_w^{ij} towards the less fit strain dependent only on the ratio of radial expansion velocities u_i/u_j [1,2] given by

$$v_w^{ij} = \sqrt{s_{ij}\left(2+s_{ij}\right)} \quad \text{with} \quad s_{ij} = 1 - u_i/u_j. \tag{S2.1}$$

Korolev et al. found that this relationship holds in *S. cerevisiae* expansions at large lengths expanded; at small lengths expanded, the prediction overestimates the wall velocity [1].

We tested if eq. (S2.1), using the average fitnesses s_{ij} of our *E. coli* strains derived from growing strains independently and listed in Table 4 of the main text (as well as on the top of Table A below), could predict the v_w^{ij} that we measured from directly tracking the growth of sectors (see the Measuring the domain wall velocities v_w^{ij} section). As shown on the top of Table A, eq. (S2.1) overestimated our measured wall velocities by a factor between 5 and 10.

As mentioned in the main text, although the rank order of our strains' expansion velocities was consistent between sets of plates, the precise values of s_{ij} varied. Therefore, to control for plate-to-plate variability, we ran another experiment where we grew the colonies used to determine u_i and u_j on the *same* plate as the colony used for evaluating v_w^{ij} from the motion of domain walls; i.e., on one plate we inoculated a colony of a fast growing strain i, a colony of a slower growing strain j, and a mixed colony composed of 10% of strain i and 90% of strain j (the ratio of strains was chosen so that single sectors of the more fit strain would form). Unsurprisingly, the radial expansion velocities of each of the three colonies at large lengths expanded was less than the velocity when grown on plates alone; we attribute this to nutrient depletion resulting from the presence of additional colonies.

Using the radial expansion velocities of the i and j expansions per plate (u_i and u_j), we calculated s_{ij} per plate and also directly measured v_w^{ij} per plate by tracking the growth of domain walls. We found that the geometrically motivated prediction of eq. (S2.1) again overestimated the magnitude of v_w^{ij} on *every* plate by almost a factor of 5. The average values of s_{ij}, the average predicted v_w^{ij}, and the average measured v_w^{ij} can be seen on the bottom of Table A. To more clearly visualize the discrepancy from this set of experiments, we used the average *predicted* v_w^{ij} to plot the expected average sector width $\langle \phi - \phi_0 \rangle$ via eq. (12) of the main text, or $\langle \phi - \phi_0 \rangle = 2v_w^{ij} \ln\left(R/R_0\right)$, and compared it to the average *experimental* sector width in Figure A. The predicted width overestimated the experimental width by over 3 standard deviations at the largest length expanded.

Both Figure A, displaying the predicted average angular width vs. the experimentally measured average width and Table A, where we compared the predicted values of v_w^{ij} to

One colony per plate

Strain	$s_{iR} = u_i/u_R - 1$	$v_w^{iR} = \sqrt{s_{iR}\,(2 + s_{iR})}$	v_w^{iR}: tracking sectors
eYFP	0.09 ± 0.03	0.43 ± 0.08	0.06 ± 0.02
eCFP	0.09 ± 0.03	0.43 ± 0.08	0.06 ± 0.02
Black	0.06 ± 0.01	0.35 ± 0.03	0.06 ± 0.02
mCherry	0	0	0

Controlling for plate-to-plate variability: three colonies per plate

Sweeper	$s_{iR} = u_i/u_R - 1$	$v_w^{iR} = \sqrt{s_{iR}\,(2 + s_{iR})}$	v_w^{iR}: tracking sectors
eYFP	0.05 ± 0.01	0.34 ± 0.03	0.06 ± 0.02
eCFP	0.06 ± 0.01	0.32 ± 0.04	0.06 ± 0.02
Black	0.03 ± 0.01	0.26 ± 0.04	0.06 ± 0.02

Table A. The predicted wall velocity from eq. (S2.1), $v_w^{ij} = \sqrt{s_{ij}\,(2 + s_{ij})}$, vs. the directly measured wall velocity from the growth of more fit sectors. *Top*: Average $s_{iR} = 1 - u_i/u_R$ from individual colonies growing on agar plates; values were taken from Table 4 of the main text. The predicted wall velocity based on the average values of s_{iR} overestimated the actual, directly measured wall velocity by a factor between 5 and 10. *Bottom*: To control for plate-to-plate variability, three colonies were grown per plate: a pure expansion of type i, a pure expansion of type j, and a mixed colony of strains i and j that was used to directly measure v_w^{ij}. We measured s_{ij} and v_w^{ij} per plate and found that the *predicted* v_w^{ij} overestimated the measured wall velocity by a factor of about 5 on *every* plate. The values in the table above are the s_{ij} and v_w^{ij} averaged over plates.

experimentally measured values, indicate that the the geometrical equation (S2.1), $v_w^{ij} = \sqrt{s_{ij}\,(2 + s_{ij})}$, predicts much larger wall velocities than we actually measure. Controlling for plate-to-plate variability does not change this conclusion.

As mentioned above, Korolev et al. [1] found that geometric predictions overestimated the wall velocity in yeast expansions for small lengths expanded. However, at large lengths, the wall velocity approached its predicted value. A similar effect is likely occurring with our *E. coli* strains, except that we do not find an approach to the predicted wall velocity value over the length of our experiments; the wall velocity was *always* less than the prediction of equation (S2.1). It is possible that unaccounted mechanical forces, such as surface or line tensions, damp the ability of more fit strains to bulge outwards, preventing geometric arguments from applying. Another possible explanation is that simple geometric arguments describing wall motion no longer hold as a colony roughens. There is also the possibility of unexpected mutualistic or antagonistic chemical secretions between strains. However, this explanation seems unlikely because our strains were isogenic besides their inserted plasmids and the mutations in the black strain; mutualistic interactions are not expected from the basal genotypes of our strains. Furthermore, the ratio of the expansion velocities s_{ij} of the three colonies grown on the same plate matched the s_{ij} of strains grown on independent plates for this batch. Order of magnitude estimates of diffusion constants suggest that mutualistic or antagonistic secretions would have diffused over the entire plate during the 8 days of an experiment and would have likely changed the relative fitnesses of the expanding colonies.

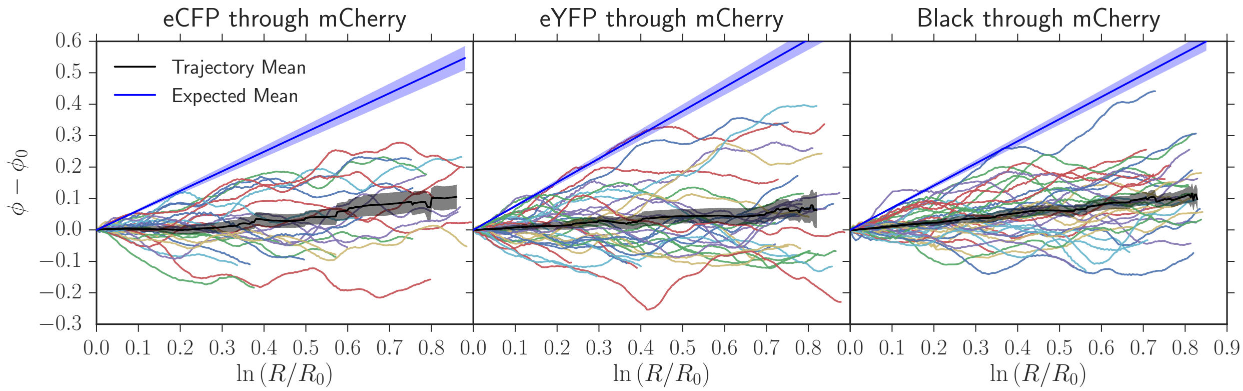

Fig A. Expected average angular growth of sectors (blue) from equation (12) using strains' average relative expansion velocities vs. the actual average angular growth (black). The shaded areas are the standard error of the mean and the colored lines are individual traces of sectors' angular width. Equation (12), using the predicted wall velocity v_w^{ij} extracted from the ratio of the strain expansion velocities in eq. (S2.1), overestimated the average angular width at the largest $\ln(R/R_0)$ by over 3 standard deviations.

In conclusion, for the *E. coli* strains and the growth conditions we used, it was not possible to predict the wall velocities from independently measured radial expansion velocities using the geometrical argument underlying eq. (S2.1). Understanding the origin of this discrepancy is an interesting avenue for future investigation. Since this work focuses on competition within colonies, we use directly measured wall velocities from both image analysis (see the Measuring v_w^{ij} section) and our two-point correlation function fitting technique (see Table 3) to predict our experiments' evolutionary dynamics.

References

1. Korolev KS, Müller MJI, Karahan N, Murray AW, Hallatschek O, Nelson DR. Selective sweeps in growing microbial colonies. Physical Biology. 2012;9(2):026008. doi:10.1088/1478-3975/9/2/026008.

2. Gralka M, Stiewe F, Farrell F, Möbius W, Waclaw B, Hallatschek O. Allele surfing promotes microbial adaptation from standing variation. Ecology Letters. 2016;19(8):889–898. doi:10.1111/ele.12625.
