# Supplementary figures and images for: Genetic drift and selection in many-allele range expansions
Source: PLoS Comput Biol. 2017 Dec 1;13(12):e1005866. doi: 10.1371/journal.pcbi.1005866 (PMC5728587; doi:10.1371/journal.pcbi.1005866)

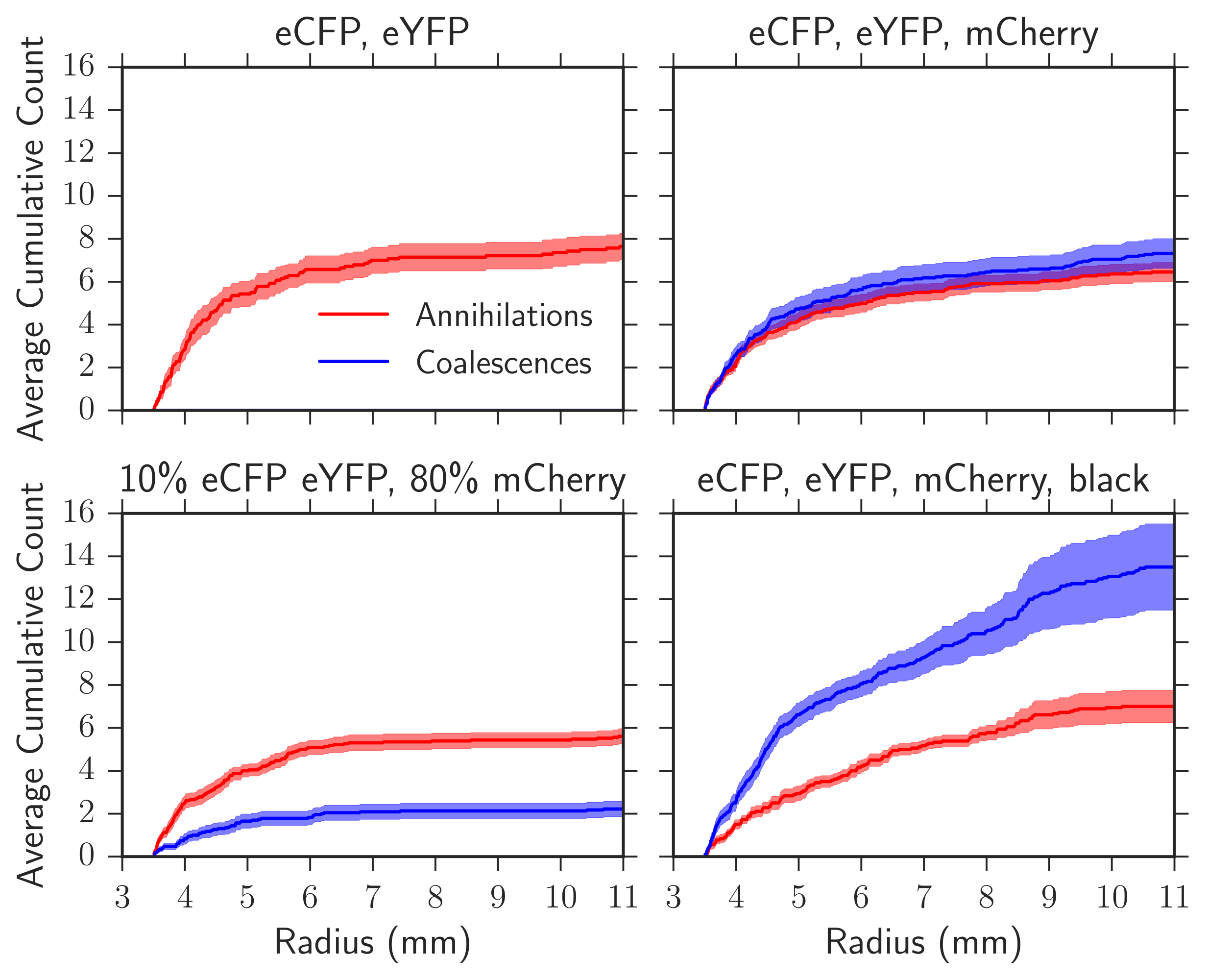

Supplement: S1 Fig — All strains were inoculated in equal fractions except for the experiment with 10% of eCFP, 10% of eYFP and 80% of mCherry. The annihilation and coalescence rates (the slope of the respective curves) decrease as radius increases as there are less domain walls due to previous collisions and also because inflation decreases the probability of two walls colliding per length expanded. As the number of colors increases, coalescences occur more often than annihilations. (TIF) [file pcbi.1005866.s003.tif]

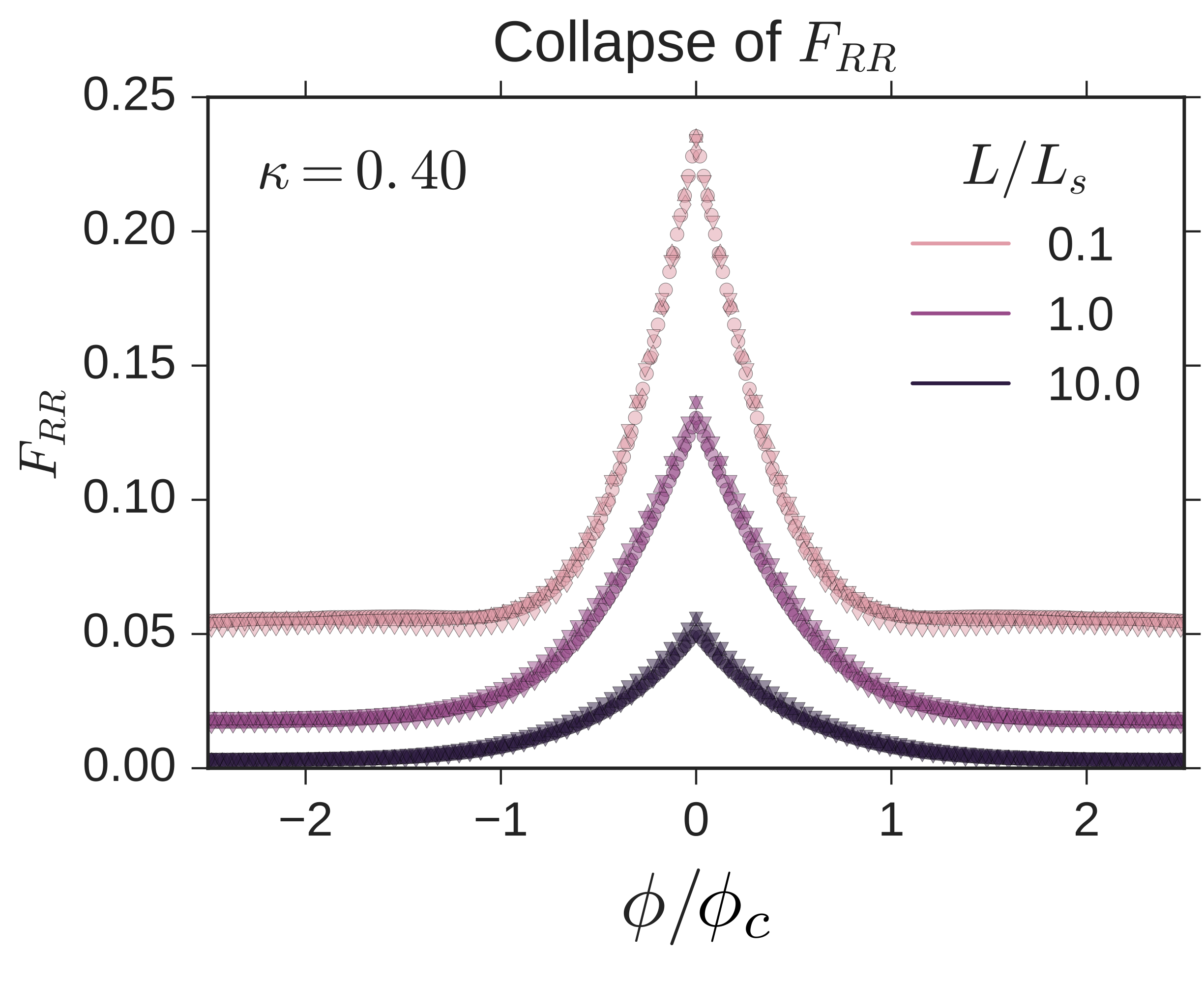

Supplement: S2 Fig — We ran four simulations where we varied vw (the velocity that the two other more fit strains swept through the less fit strain) and R0 such that Ls=Dw/vw2 changed but κ=R0/Ls was fixed. Each simulation has a different symbol in the plot. We found that Fij could be collapsed at the same L/Ls as long as κ remained fixed (we arbitrarily set it to κ = 0.4) and as long as the angular variable ϕ was rescaled by ϕc=8Dw/R0. If ϕc approached the system size ϕc ≈ 2π, Fij could not be collapsed onto the above curves due to finite size effects. Note that even though we only show FRR, all correlation functions Fij could be collapsed using this procedure. (TIF) [file pcbi.1005866.s004.tif]

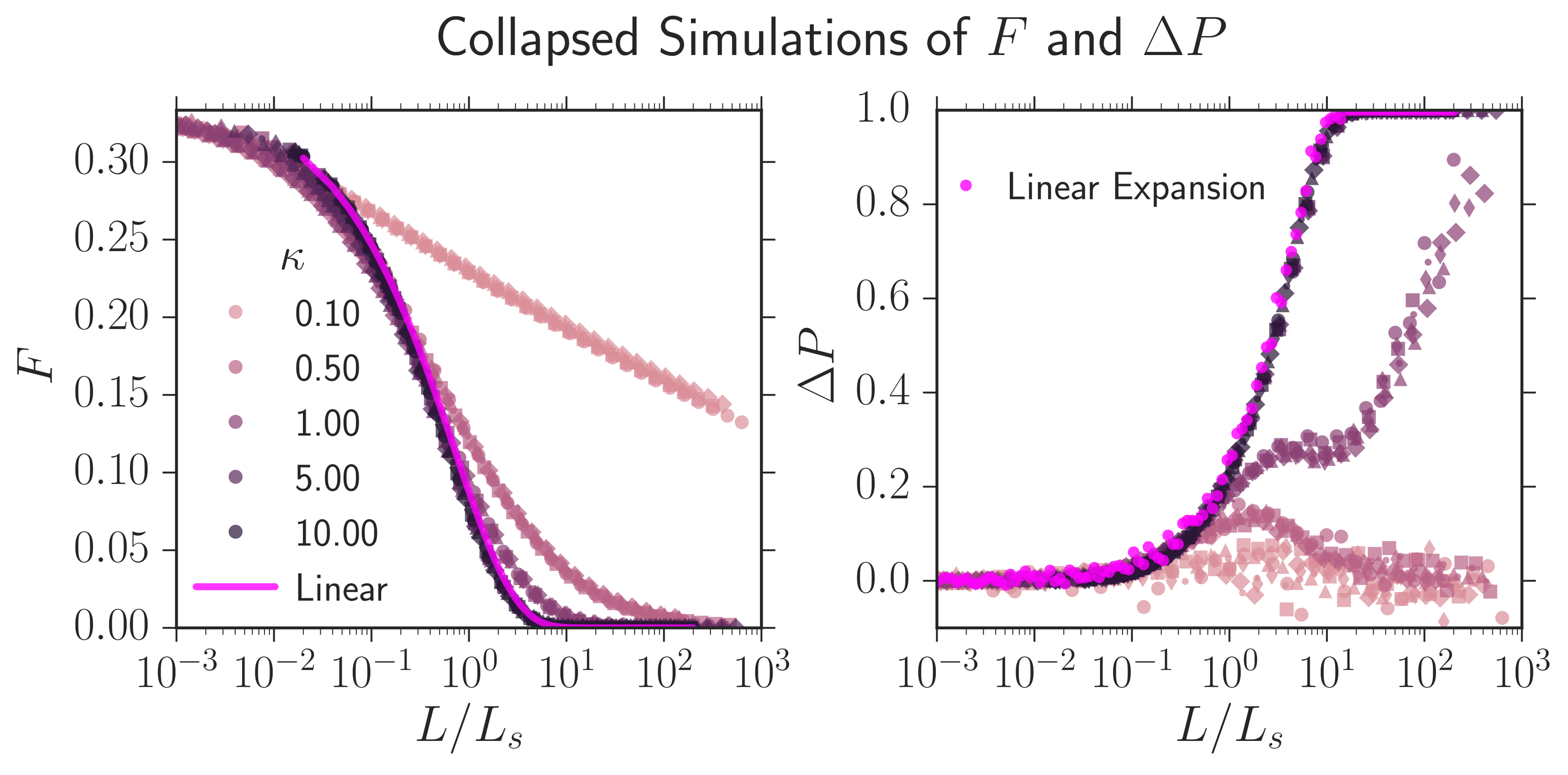

Supplement: S3 Fig — Identical to Fig 6 except the y-axis of F(L/Ls, κ) is placed on a linear scale, which may be useful for comparison with experiments. (TIF) [file pcbi.1005866.s005.tif]

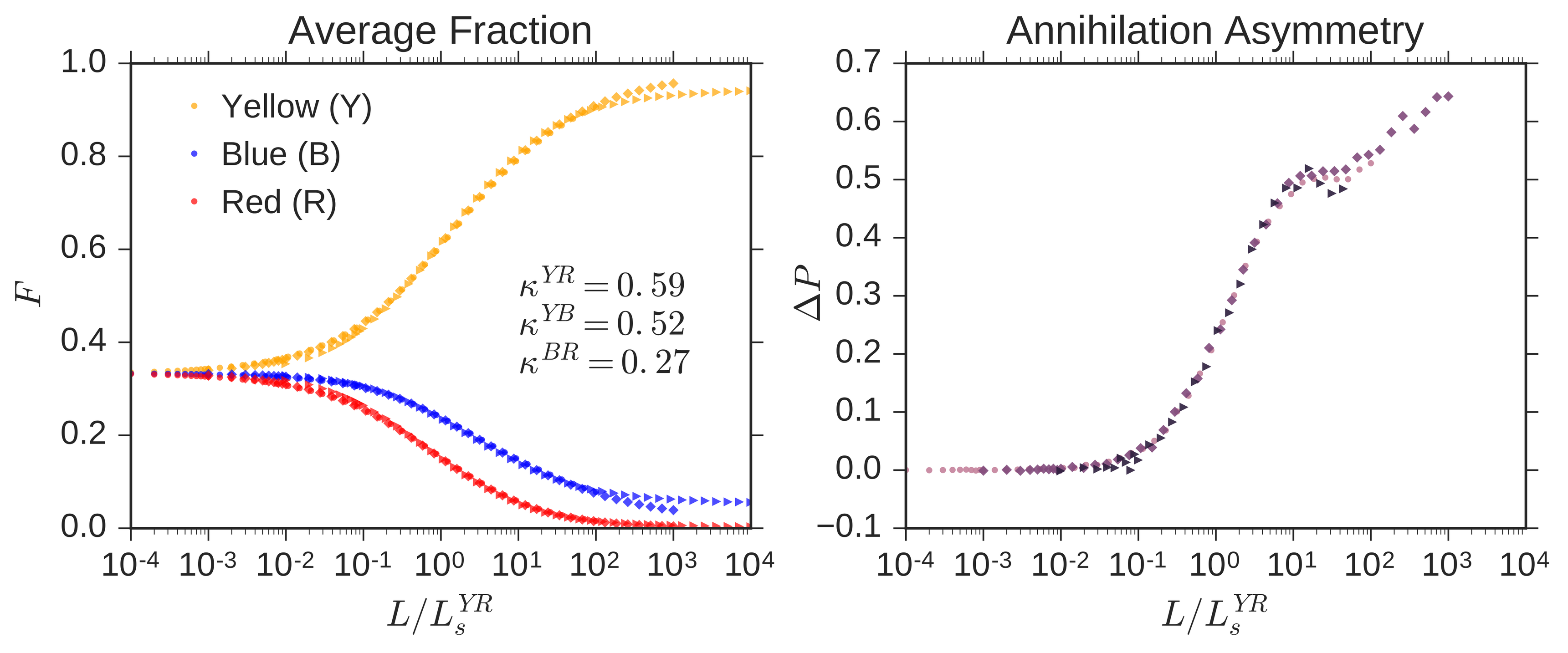

Supplement: S4 Fig — We simulated three competing strains and arbitrarily chose a fixed set of κij=R0/Lsij=R0(vwij)2/Dw to reflect differing selective advantages between the strains. We set the initial radius of the expansion R0 = N0a/(2π) to N0 = {200, 2000, 20000} in three simulations and altered vwij to tune the set of κij’s to their fixed values. We found that the dynamics collapsed as long as L was rescaled by any selection length scale in the system, i.e. L/Lsij (we chose to use LsYR). The diamond simulation, corresponding to N0 = 200, deviated slightly from the other simulations because of finite size effects (i.e. when ϕc ∼ 2π). (TIF) [file pcbi.1005866.s006.tif]

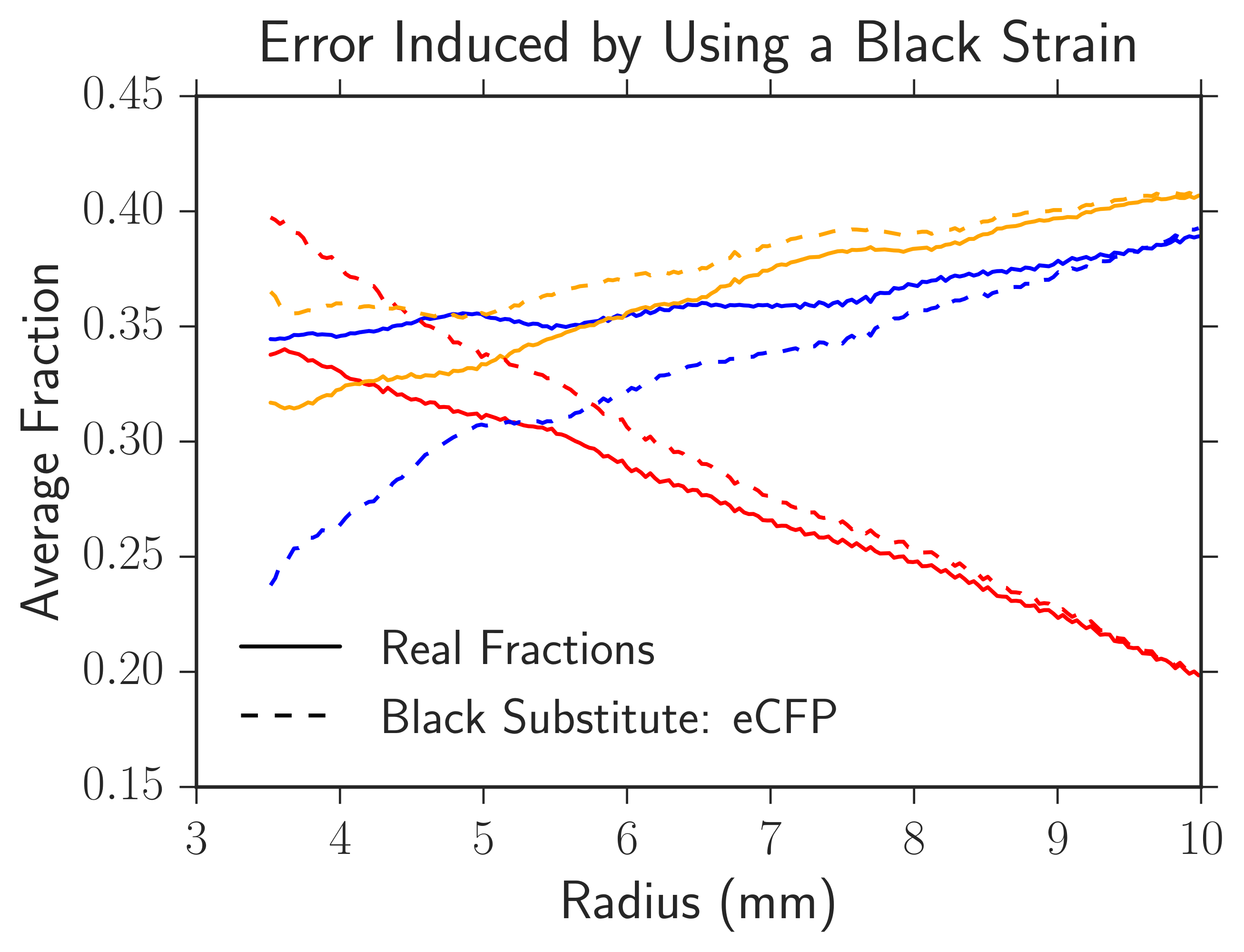

Supplement: S5 Fig — To estimate the image analysis artifacts introduced by using a non-fluorescent, black strain we performed an experiment with three fluorescent strains (eCFP, eYFP, and mCherry in equal initial proportions) and analyzed the data twice: once where we included all three fluorescent channels and once where we excluded the eCFP channel and treated it as if it were a black strain. We compared the black-substituted average fractions Fi (the dashed lines) to the real fractions as a function of radius (the solid lines). At a small radius relative to R0 = 3.5 mm, the error from introducing a black strain was large; this is likely because we defined black as the absence of any other channels and channels typically had large overlaps close to the homeland. At large radius, the error from introducing a black strain was negligible. (TIF) [file pcbi.1005866.s007.tif]

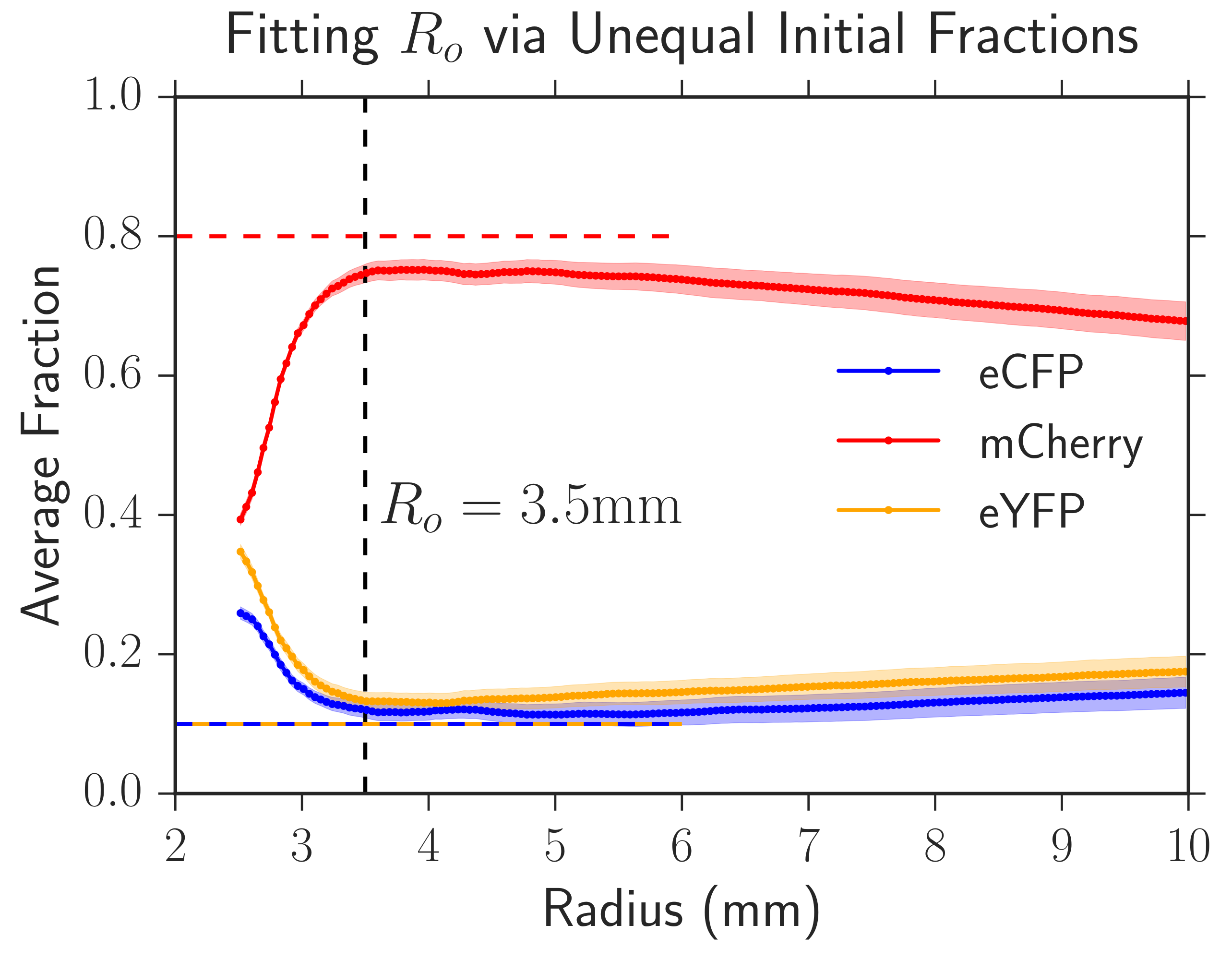

Supplement: S6 Fig — To fit the radius R0 where our image analysis package became accurate, we inoculated 80% of mCherry, 10% of eCFP, and 10% of eYFP in 10 range expansions and tabulated the average fraction of each strain. The inoculated fractions are illustrated by dashed lines. As seen in the plot, at a radius of approximately R0 = 3.50 ± 0.05 mm the measured average fractions were closest to the inoculated fractions. Our image analysis package inaccurately predicted fractions in the homeland because of significant overlap between the strains. (TIF) [file pcbi.1005866.s008.tif]

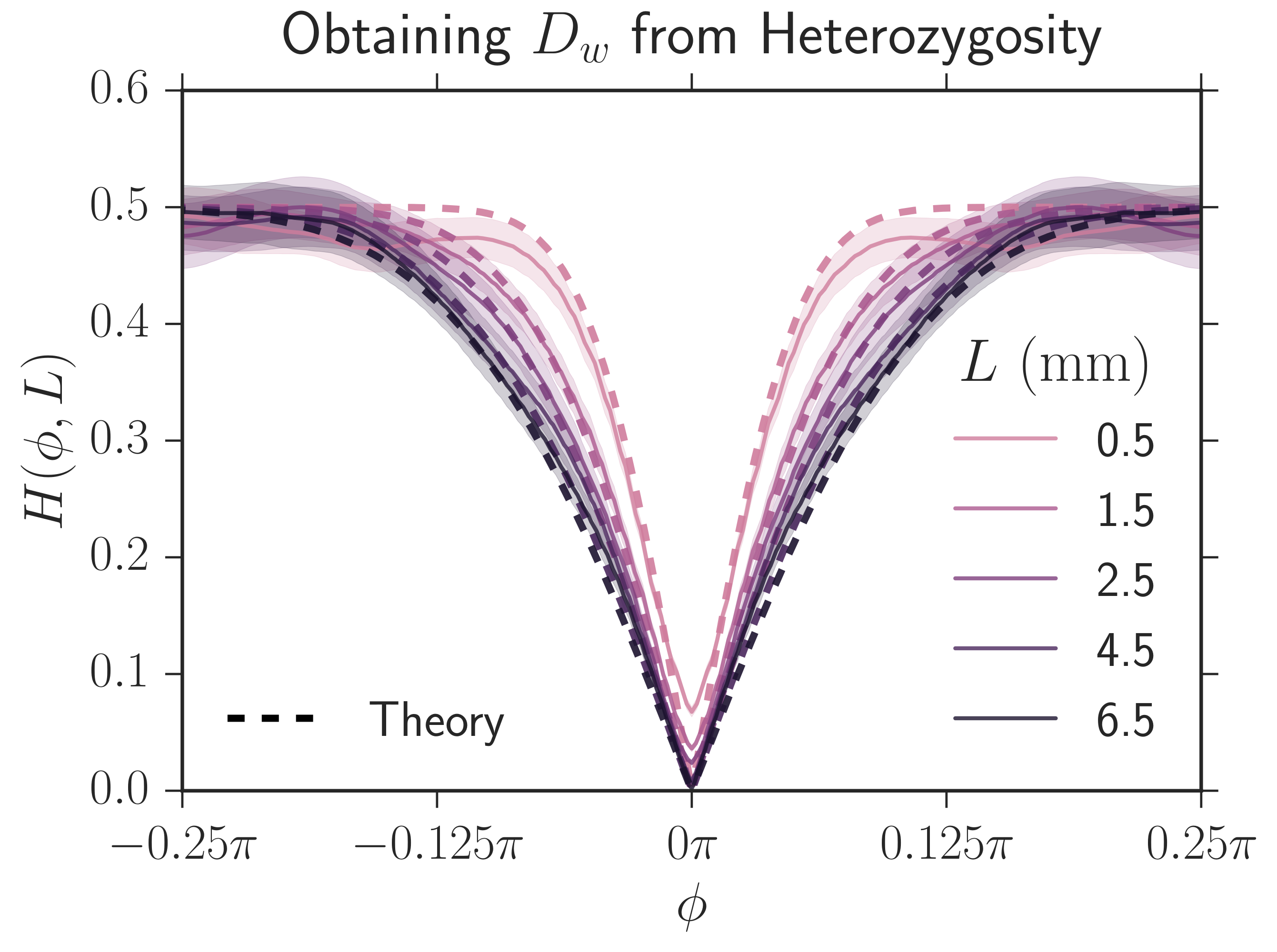

Supplement: S7 Fig — The same as the right side of Fig 12 except with error bars; the shaded areas are the standard error of the mean. (TIF) [file pcbi.1005866.s009.tif]

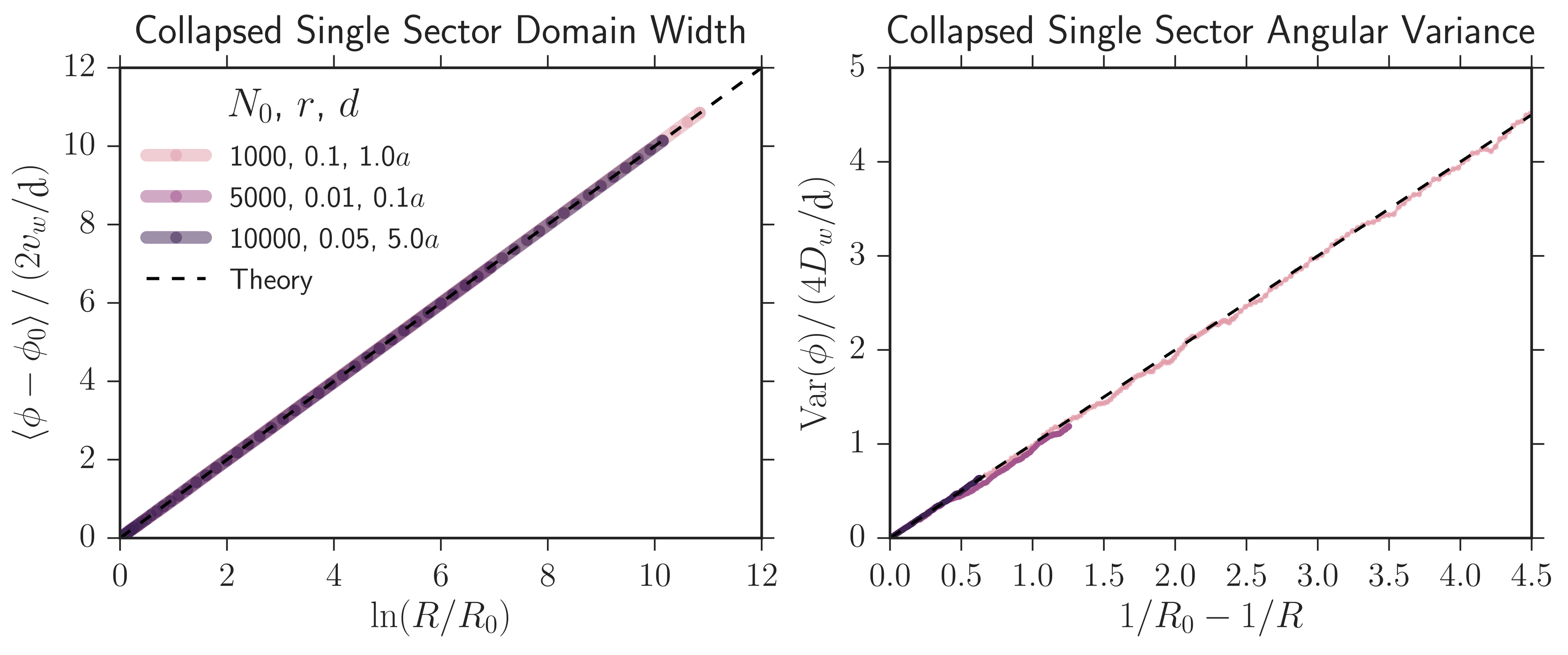

Supplement: S8 Fig — We simulated a single fit sector sweeping through a less fit strain. It is expected that the fit strain sector dynamics satisfy 〈ϕ − ϕ0〉 = 2vw ln(R/R0) and Var(ϕ) = 4Dw(1/R0 − 1/R), as seen in S1 Appendix. To test that our simulation appropriately reproduced this behavior, we quantified the average angular growth 〈ϕ − ϕ0〉 and angular variance Var(ϕ) as we varied the simulation parameters N0 (initial number of cells), r (selective advantage of the fitter strain), and d (distance the colony expanded each generation). The cell width, a, was kept constant. These parameters relate to the sector dynamics via Dw = a2/(2d), vwij=arij/d, and R0 = (N0a)/(2π). We confirmed that both the average angular growth 〈ϕ − ϕ0〉 and angular variance Var(ϕ) had the correct functional form and dependence on the microscopic parameters (the dashed black line). In the main text, we used d = a for simplicity. (TIF) [file pcbi.1005866.s010.tif]
